# Interleukin 11–Induced MicroRNAs as Functional Mediators and Circulating Biomarkers of Cardiac Fibrosis

**DOI:** 10.1161/CIRCRESAHA.125.326799

**Published:** 2026-03-17

**Authors:** Roman Tikhomirov, Veronika Boichenko, Benedict Reilly-O’Donnell, Carla Lucarelli, Prashant Kumar Srivastava, Maryam Anwar, Chi Him Kendrick Yiu, Julia Dielesen, Victoria Maria Noakes, Santiago Nicolas Piella, Diana Kazharova, Mathilde Labbé, Zoe Kwan, Germana Zaccagnini, Catherine Mansfield, Maddalena Tessari, Lorenzo Menicanti, Simona Greco, Mark Sweeney, Joseph Okafor, Przemysław Leszek, Giuseppe Faggian, Giovanni Battista Luciani, Costanza Emanueli, Fabio Martelli, Julia Gorelik

**Affiliations:** 1 National Heart and Lung Institute (R.T., V.B., B.R.-O., C.L., P.K.S., M.A., C.H.K.Y., J.D., V.M.N., D.K., M.L., Z.K., C.M., J.O., C.E., J.G.), Imperial College London, United Kingdom.; 2 Medical Research Council (MRC)-Laboratory of Medical Sciences (M.S.), Imperial College London, United Kingdom.; 3 Molecular Cardiology Laboratory, Istituto di Ricovero e Cura a Carattere Scientifico (IRCCS) Policlinico San Donato, Milan, Italy (R.T., V.B., S.N.P., G.Z., S.G., F.M.).; 4 Department of Surgery, Dentistry, Pediatrics, and Gynecology, The University of Verona, Italy (R.T., V.B., C.L., M.T., G.F., G.B.L.).; 5 Cardinal Wyszynski National Institute of Cardiology, Warsaw, Poland (P.L.).; 6 Department of Cardiac Surgery, Istituto di Ricovero e Cura a Carattere Scientifico (IRCCS) Policlinico San Donato, Milan, Italy (L.M.).; 7 Imperial College Healthcare National Health Service (NHS) Foundation Trust, Hammersmith Hospital, London, United Kingdom (J.O.).

**Keywords:** aortic valve stenosis, biomarkers, fibrosis, microRNAs, translational research, biomedical

## Abstract

**BACKGROUND:**

Cardiac fibrosis can be triggered by several pathologies, including ischemic heart disease and aortic stenosis. Cardiac fibrosis is brought about by uncontrolled ECM (extracellular matrix) deposition by myofibroblasts. IL (interleukin)-11 (*Il11*) has been demonstrated as a trigger of multiorgan fibrosis. However, the molecular mechanisms underpinning IL-11–induced fibrosis require further characterization. Recent studies indicate that microRNA dysregulation contributes to the pathogenesis of cardiac fibrosis and can be targeted therapeutically. This study explored the hypothesis that microRNAs act as downstream effectors of IL-11–induced cardiac fibrosis. Moreover, we investigated the translational potential of IL-11–regulated microRNAs as circulating biomarkers of cardiac fibrosis in patients with aortic stenosis.

**METHODS:**

A bioinformatic microRNA target prediction analysis was used to identify candidate microRNAs regulated by IL-11. Experimental validation was performed in cardiac fibroblasts from postinfarction failing and healthy rat hearts, after IL-11 stimulation. Functional studies assessed the effects of microRNA modulation on fibrotic gene expression in cardiac fibroblasts using microRNA inhibitor–based and mimic-based transfection. Bioinformatic analysis and luciferase assay identified candidate microRNA targets downstream of IL-11. Findings were further evaluated in transverse aortic constriction and cardiomyocyte-specific *Il11*-overexpression (Tg-Il11 [transgenic mouse model with cardiomyocyte-specific *Il11* overexpression]) mouse models and in left ventricular tissue, peripheral plasma, and plasma extracellular vesicles from patients with aortic stenosis.

**RESULTS:**

MicroRNA-27b-5p and microRNA-497-5p were identified as novel downstream effectors of IL-11 signaling. IL-11 increased the expression of both microRNAs in cardiac fibroblasts; transfection with either microRNA inhibitor reduced, whereas microRNA mimics increased, profibrotic mRNA levels. Furthermore, microRNA-27b-5p and microRNA-497-5p converged on HIF (hypoxia-inducible factor)-1 signaling by targeting its regulator EGLN (Egl-9 family hypoxia-inducible factor). Increased microRNA levels were observed alongside reduced expression of Egln1 and Egln2 in 2 mouse models. In patients with aortic stenosis, myocardial and circulating levels of these microRNAs correlated with the severity of left ventricular fibrosis, indicating these microRNAs’ potential as new circulating biomarkers of cardiac fibrosis.

**CONCLUSIONS:**

In this study, we have newly identified the potential value of microRNA-27b-5p and microRNA-497-5p as actionable biomarkers of the profibrotic response to IL-11 in the heart. Future studies should validate the translational potential of the microRNAs as new clinical biomarkers and therapeutic targets.

**Novelty and Significance**

**What Is Known?**

- Cardiac fibrosis contributes to adverse remodeling and heart failure in conditions such as ischemic heart disease and aortic stenosis.
- IL (interleukin)-11 is a key driver of fibrotic responses in multiple organs, including the heart.
- MicroRNAs are important regulators of gene expression and have been implicated in the development of cardiac fibrosis.

**What New Information Does This Article Contribute?**

- This study identifies microRNA-27b-5p and microRNA-497-5p as novel downstream effectors of IL-11–driven cardiac fibrosis.
- The 2 microRNAs promote fibrotic gene expression by converging on hypoxia-inducible factor 1 signaling through regulation of EGLN mRNA expression.
- Circulating levels of these microRNAs reflect the severity of cardiac fibrosis in patients with aortic stenosis, highlighting their potential as biomarkers.

Cardiac fibrosis drives adverse remodeling and progression to heart failure, yet the molecular links between profibrotic signaling and gene regulation remain incompletely defined. This study identifies 2 microRNAs acting downstream of IL-11 that promote extracellular matrix production through hypoxia-related pathways. Importantly, levels of these microRNAs in cardiac tissue and circulation correlate with fibrosis severity in patients with aortic stenosis, highlighting their potential as biomarkers and therapeutic targets.

**Meet the First Author, see p e000752**

Cardiac fibrosis is a pathological condition that accompanies various heart diseases.^1^ It impairs heart function by extensive deposition of ECM (extracellular matrix) proteins, especially collagens, into the myocardium.^2^ ECM remodeling increases heart stiffness, driving heart failure (HF).^3^ Cardiac fibroblasts (CFs) are the primary source of ECM proteins. Activated fibroblasts, or myofibroblasts, can be characterized by α-SMA (α-smooth muscle actin) stress fibers and increased expression of profibrotic factors. They play a key role in cardiac fibrosis by participating in ECM reorganization and potentiating further fibroblast activation.^4^ CFs can be activated by TGFβ1 (transforming growth factor β1), which induces fibrosis-related gene transcription through the SMAD (small mothers against decapentaplegic) cascade.^5^ Schafer et al^6^ revealed that exogenous TGFβ1 supplementation increased the levels of IL (interleukin)-11 in primary CFs. Further experiments showed that the activation of the IL11Rα receptor provokes a strong profibrotic state in CFs through ERK (extracellular regulated kinase) phosphorylation, transcriptional regulation of collagens, and autocrine IL-11 production. Accordingly, *IL-11ra* (interleukin 11 receptor subunit alpha) knockout mice were protected from developing fibrosis.^6^ IL-11 has been shown to be elevated in distinct cardiac conditions linked to ECM remodeling, such as myocardial infarction (MI)^7^ and pressure overload.^8^ Plasma levels of IL-11 have also been shown to be increased in congestive HF patients.^9^ Moreover, increased IL-11 was detected in the aortic tissue and plasma of patients with acute thoracic aortic dissection.^10^ These studies candidate IL-11 as a therapeutic target and biomarker of cardiac fibrosis. Notwithstanding, the downstream effectors and noncanonical signaling pathways triggered by IL-11 in CFs remain largely unexplored.^11^ One class of molecules that could regulate the action of IL-11 in CFs is microRNAs. The canonical action of individual microRNAs consists of posttranscriptional repression of gene expression, achieved by their binding to a pool of specific mRNA to induce their degradation or translational inhibition.^12^ Several microRNAs have already been shown to be relevant for human cardiovascular disease and cardiac fibrosis^12–15^ and to mediate the fibrotic response triggered by TGFβ1.^16–18^ Moreover, microRNAs have been proposed to have value as circulating biomarkers of organ fibrosis,^19^ especially when circulating in small extracellular vesicles (EVs).^20^ The involvement of microRNAs in the heart’s fibrotic response to IL-11 remains unexplored.

Aortic stenosis (AS) is caused by the progressive calcification and reduced mobility of the aortic valve leaflets, leading to pressure overload on the left ventricular (LV), hypertrophic, and fibrotic remodeling. AS is the most frequent heart valve disease in the Western world, with more than 2% of patients over 60 years suffering from this condition.^21^ Treatments for AS include surgical aortic valve replacement or transcatheter aortic valve implantation. If left untreated, the mortality rate associated with AS reaches 50% at 1 year after symptom onset.^22^ Current guidelines recommend treatment for AS in symptomatic patients or in cases with LV impairment. However, symptoms are challenging to assess in this cohort of patients. Recent imaging studies have demonstrated that even a minor LV impairment is associated with worse outcomes after surgical aortic valve replacement or transcatheter aortic valve implantation,^23,24^ and LV fibrosis is associated with increased long term mortality despite treatment.^25^ Therefore, imaging and laboratory biomarkers enabling the estimation of fibrosis in the LV would crucially aid in planning the timing of intervention in these patients, to prevent subtle yet significant myocardial damage and maladaptive remodeling, ultimately improving outcomes. Identifying compelling laboratory biomarkers associated with cardiac fibrosis has the potential to improve our ability to determine the optimal time of intervention in patients with severe AS. In this study, we have combined in silico, in vitro, and in vivo studies, along with clinical samples from a highly selected population of patients with severe AS. This has led us to newly identify and assess the potential value of microRNA-27b-5p and microRNA-497-5p as biomarkers of cardiac fibrosis.

## Methods

### Data Availability

The data that support the findings of this study are available from the corresponding author on reasonable request. For full details of methods, please see the Supplemental Material.

### Ethics Regulation of Work on Human and Animal Samples

Studies on human tissue (AS cohorts, healthy volunteer blood, donor heart tissue) followed the principles outlined in the Helsinki Declaration and the Italian and Polish laws and guidelines and were authorized by local ethics committees (for Italy: Protocol No. 2438, January 27, 2009 and CE No. 85/int/2016 June 9, 2016; for Poland Protocol No. IK-NPIA-0021-14/1426/18). The use of human donor hearts for preparation of CFs was approved under Research Ethics Commitee (REC) reference 19/SC/0257; Integrated Research Application System (IRAS) project ID: 264059.

Human blood samples used in the AS validation cohort were obtained from the Imperial College Healthcare Tissue Bank. Imperial College Healthcare Tissue Bank is supported by the National Institute for Health Research Biomedical Research Centre based at Imperial College Healthcare National Health Service (NHS) Trust and Imperial College London. Imperial College Healthcare Tissue Bank is approved by Wales REC3 to release human material for research (22/WA/0214), and the samples for this project (R20040) were issued from subcollection reference number NHL_BR_19_007.

The work on the mouse transverse aortic constriction (TAC) models was performed at the San Raffaele Hospital research institute (Milan) after approval by both the Italian National Institutes of Health and the local Institutional Animal Care and Use Committee. All experimental procedures complied with the Guidelines of the Italian National Institutes of Health and the *Guide for the Care and Use of Laboratory Animals* (Institute of Laboratory Animal Resources, National Academy of Sciences, Bethesda, MD).

The Tg-Il11 (transgenic mouse model with cardiomyocyte-specific *Il11* overexpression) model was performed in the United Kingdom under the UK Home Office Project License P108022D1 and under the approval and supervision of the Imperial College Animal Welfare Ethical Review Body, and according to the Animals (Scientific Procedures) Act 1986 Amendment Regulations 2012, incorporating the EU directive 2010/63/EU.

MI and sham rat surgical procedures and perioperative management were performed in accordance with the United Kingdom Home Office Guide on the Operation of the Animals (Scientific Procedures) Act 1986 and EU Directive 2010/83, under the approval of the animal welfare and ethics review board of Imperial College London.

## Results

### CFs Are Activated on In Vitro Treatment With IL-11 and in the Post-MI Failing Heart

**Figure 1.**
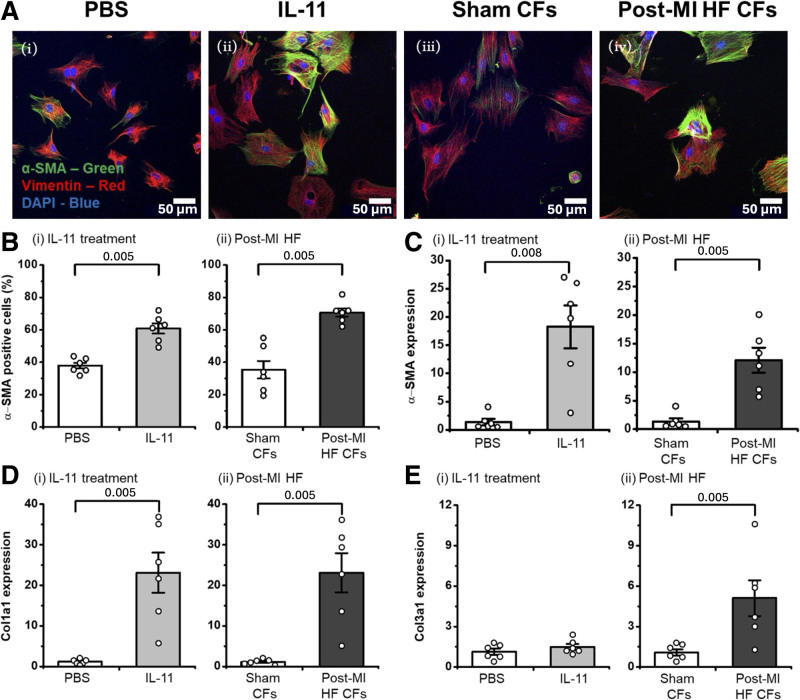
**Characterization of IL (interleukin)-11 and post–myocardial infarction (MI) heart failure (HF) models of fibrosis by immunostaining and RT-qPCR. A**, Representative images of rat cardiac fibroblasts (rCFs) stained for α-SMA (α-smooth muscle actin; green), vimentin (red), and DAPI (4′,6-diamidino-2-phenylindole; blue): rCFs treated with (**i**) PBS; (**ii**) IL-11 (5 ng/mL, 24 hours); or harvested from (**iii**) sham-operated or (**iv**) post-MI HF rat; **B** through **E**, experiments realized for the 2 protocols: (**i**) in vitro stimulation with IL-11 and (**ii**) harvesting from the failing and sham-operated hearts. **B**, Quantification of α-SMA–positive cells (%; N=6, n=2). ≈200 cells were quantified for each condition, with 10 cells per image. Reverse transcription quantitative PCR (RT-qPCR) analysis showing (**C**) α-SMA, (**D**) Col1a1 (type I alpha[1] collagen), and (**E**) Col3a1 (type III alpha[1] collagen) mRNA expression (normalized to GAPDH; N=6; n=3). The Mann-Whitney *U* test was used to determine significant differences between groups, with *P* values shown. CF indicates cardiac fibroblast.

Administration of IL-11 to cell cultures with high fibroblast purity promoted differentiation of rat cardiac fibroblasts (rCFs) into α-SMA–positive myofibroblasts (Figure 1Ai, 1Aii, and 1Bi). Moreover, α-SMA–positive cells were abundant in cells prepared from rat failing hearts (Figure 1Aiii, 1Aiv, and 1Bii; Figure S3). Increased expression of α-SMA (Figure 1C) was observed in rCFs treated with IL-11 or derived from post-MI failing rat hearts.

In addition, elevated Col1a1 (type I alpha[1] collagen), Col3a1 (type III alpha[1] collagen), and Mmp2 (matrix metalloproteinase-2) mRNA expression levels were detected in CFs prepared from the rat failing hearts (Figure 1Dii and 1Eii; Figure S4Aii). CFs stimulated with IL-11 in vitro demonstrated increased levels of Col1a1 and Mmp2 (Figure 1Di; Figure S4Ai), but not Col3a1 (Figure 1Ei).

### MicroRNA-497-5p and MicroRNA-27b-5p Expression Is Increased in rCFs by IL-11 and in Post-MI rCFs

**Figure 2.**
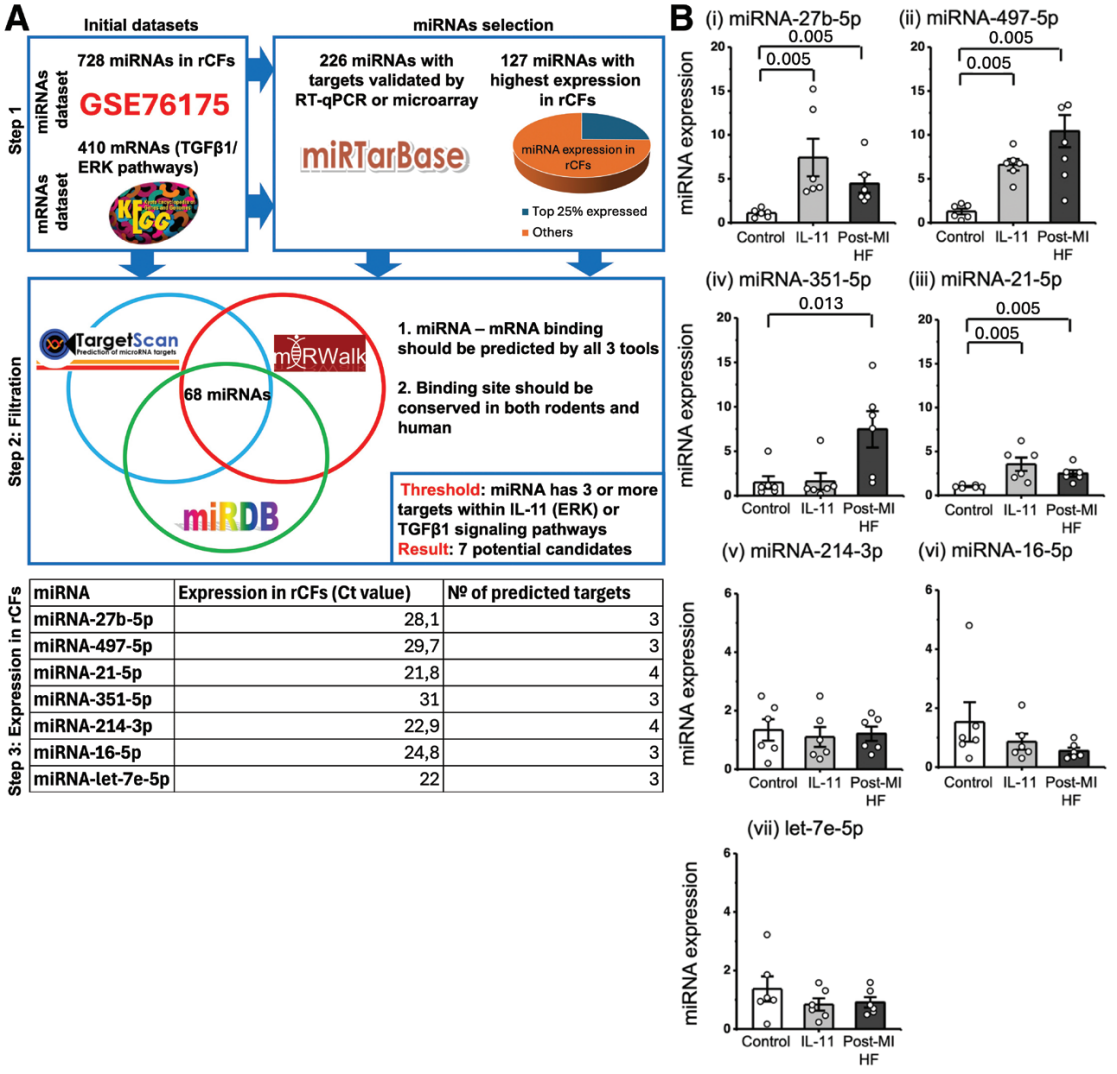
**Prediction and validation experiments for microRNAs (miRNAs) expressed in cardiac fibroblasts and associated with cardiac fibrosis through regulation of IL (interleukin)-11 and TGFβ1 (transforming growth factor β1) pathways. A**, Bioinformatic pipeline to identify the miRNAs that target mRNAs associated with Kyoto Encyclopedia of Genes and Genomes (KEGG) for the IL-11 (ERK [extracellular regulated kinase]) and TGFβ1 profibrotic pathways. **B**, Reverse transcription quantitative PCR (RT-qPCR) analysis, miRNA expression normalized to U6 in control rat cardiac fibroblasts (rCFs) (white bar), rCFs treated with IL-11 (light gray) and in post–myocardial infarction (MI) heart failure (HF) rCFs (gray): (**i**) miRNA-27b-5p, (**ii**) miRNA-497-5p, (**iii**) miRNA-21-5p, (**iv**) miRNA-351-5p, (**v**) miRNA-214-3p, (**vi**) miRNA-16-5p, and (**vii**) let-7e-5p (N=6, n=3). The Mann-Whitney *U* test was used to determine significance between the control and the group of interest, with *P* values shown.

Our bioinformatic target prediction analysis (Figure 2A) identified 68 candidate microRNAs that were predicted as modulators of the IL-11 and TGFβ1 profibrotic response. We then identified the top 7 most robust microRNA candidates (Figure 2B). These 7 microRNAs were further assessed by polymerase chain reaction of IL-11-treated rCFs and in rCFs derived from failing hearts. MicroRNA-27b-5p (Figure 2Bi), microRNA-497-5p (Figure 2Bii), and microRNA-21-5p (Figure 2Biii) were upregulated in both profibrotic stimuli: IL-11 and HF conditions. Because microRNA-21-5p has already been widely studied in the context of cardiac fibrosis,^16^ we focused our study on the remaining microRNA-27b-5p and microRNA-497-5p.

### MicroRNA-27b-5p and MicroRNA-497-5p Are Profibrotic in Rat CFs

**Figure 3.**
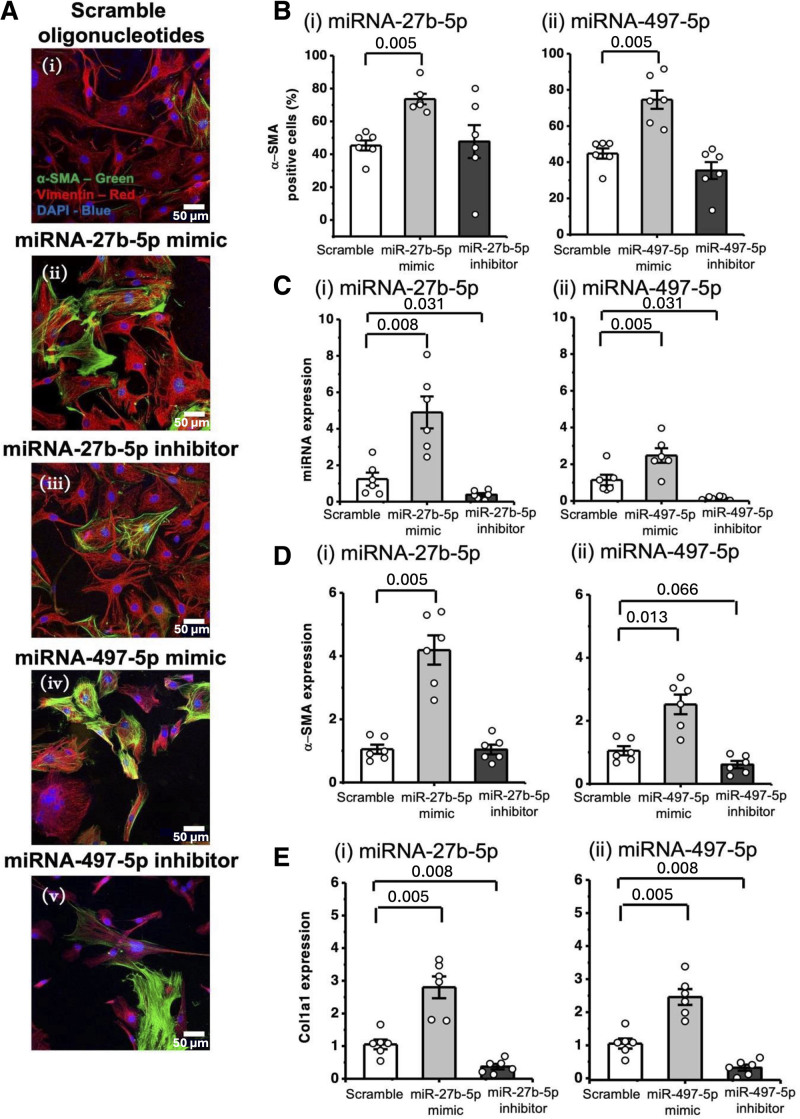
**MicroRNA (miRNA)-27b-5p and miRNA-497-5p induce profibrotic changes in cardiac fibroblasts.** Transfection of healthy rat cardiac fibroblasts (rCFs) with scramble nucleotides (white bar), miRNA-27b-5p or miRNA-497-5p mimic (labeled as miR-27b-5p or miR-497-5p mimic; light gray), or inhibitor (labeled as miR-27b-5p or miR-497-5p inhibitor; dark gray) at 5 nmol/L concentration. **A**, Representative images (**i** through **v**) stained after 24 hours with vimentin (red), α-SMA (α-smooth muscle actin; green), and DAPI (4′,6-diamidino-2-phenylindole; blue). **B** through **E**, Results of gain-of-function and loss-of-function experiments: (**i**) miRNA-27b-5p and **ii**) miRNA-497-5p. **B**, Quantification of α-SMA–positive cells (%; N=6, n=2). Approximately 300 cells were quantified per condition, with 10–20 cells per image. **C** through **E**, Reverse transcription quantitative PCR (RT-qPCR) analysis of miRNA/mRNA of interest: miRNA expression was normalized to U6 RNA expression and the scramble condition, whereas mRNA expression was normalized to UBC (ubiquitin C). Expression of (**C**) miRNA-27b-5p and miRNA-497-5p, (**D**) α-SMA and (**E**) Col1a1 (type I alpha[1] collagen; N=6, n=3). The Mann-Whitney *U* test was used to determine significance between the control and the group of interest, with *P* values shown.

To evaluate the functional role and significance of microRNA-27b-5p and microRNA-497-5p, we manipulated their expression in rCFs isolated from healthy hearts. Increased expression of either microRNA, using microRNA mimics, increased the percentage of α-SMA–positive cells (Figure 3Ai through 3Av and 3B). Transfection of rCFs with specific microRNA mimics and inhibitors successfully altered the expression of the corresponding microRNAs (Figure 3C). microRNA mimics for microRNA-27b-5p and microRNA-497-5p increased RNA expression of α-SMA, Col1a1, and Mmp2 (Figure 3D and 3E; Figure S4Bi and S4Ci). Conversely, incubation of cells with the corresponding microRNA inhibitors significantly reduced Col1a1 expression (*P*=0.008—either inhibitor versus scramble; Mann-Whitney *U* test), but not α-SMA or Mmp2 (Figure 3D and 3E; Figure S4Bi and S4Ci).

To further investigate the role of microRNA-27b-5p and microRNA-497-5p in IL-11-mediated fibrosis, rCFs were pretreated with the corresponding microRNA inhibitors or scramble nucleotides before IL-11 stimulation. Pretreatment with either microRNA inhibitor significantly reduced the percentage of α-SMA–positive cells compared with scramble +IL-11 group (microRNA-27b-5p inhibitor +IL-11, *P*=0.040; microRNA-497-5p inhibitor +IL-11, *P*=0.022; Figure S5Ai through S5Aiv and S5B). Consistent with effective inhibition, microRNA-27b-5p and microRNA-497-5p levels were reduced in IL-11–treated cells pretreated with the inhibitors compared with IL-11–treated cells pretreated with scramble nucleotides (Figure S5C). Inhibition of either microRNA also significantly attenuated IL-11–induced expression of profibrotic genes, including α-SMA (*P*=0.022 and *P*=0.042), Col1a1 (*P*=0.038 and *P*=0.044), Postn (periostin) (*P*=0.021 and *P*=0.017), and Adamts5 (a disintegrin and metalloproteinase with thrombospondin motifs 5) (*P*=0.045 and *P*=0.021), compared with scramble +IL-11 (Figure S5Di through S5Div). Statistical significance was assessed by ANOVA with a Tukey post hoc test.

**Figure 4.**
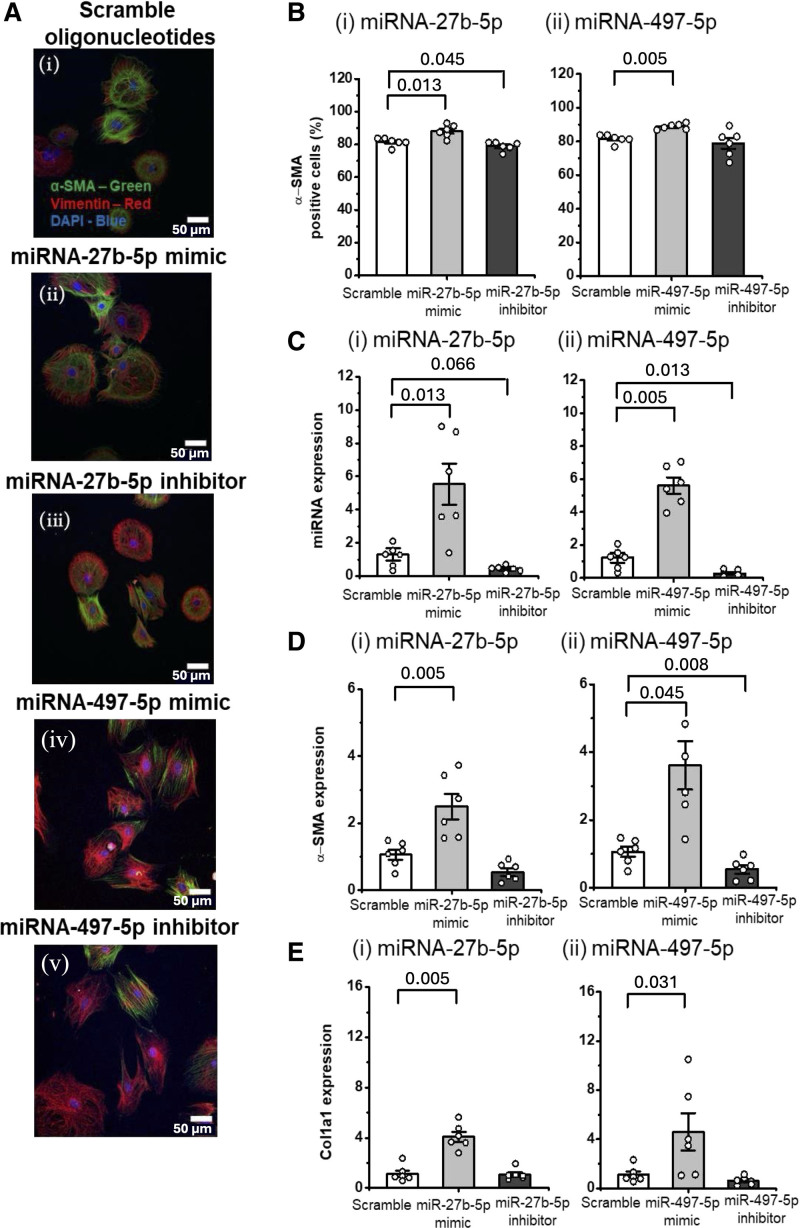
**MicroRNA (miRNA)-27b-5p and miRNA-497-5p modulation induces profibrotic changes in cardiac fibroblasts from failing hearts.** Transfection of post–myocardial infarction heart failure rat cardiac fibroblasts (rCFs) with scramble nucleotides (white bar), miRNA-27b-5p or miRNA-497-5p mimic (labeled as miR-27b-5p or miR-497-5p mimic; light gray), or inhibitor (labeled as miR-27b-5p or miR-497-5p inhibitor; dark gray) at 5 nmol/L concentration. **A**, Representative images (**i** through **v**) stained after 24 hours with vimentin (red), α-SMA (α-smooth muscle actin; green), and DAPI (4′,6-diamidino-2-phenylindole; blue). **B** through **E**, Results of gain-of-function and loss-of-function experiments: (**i**) miRNA-27b-5p and (**ii**) miRNA-497-5p. **B**, Quantification of α-SMA–positive cells (%; N=6, n=2). Approximately 100 cells were quantified for each condition, with 5 cells per image. **C** through **E**, Reverse transcription quantitative PCR (RT-qPCR) analysis of miRNA/mRNA of interest: miRNA expression was normalized to U6 RNA expression and to the scramble condition, while mRNA expression was normalized to UBC (ubiquitin C). Expression of (**C**) miRNA-27b-5p and miRNA-497-5p, (**D**) α-SMA and (**E**) Col1a1 (type I alpha[1] collagen; N=6, n=3). The Mann-Whitney *U* test was used to determine significance between the control and the group of interest, with *P* values shown.

Next, we examined how microRNA overexpression and inhibition affected rCFs derived from failing hearts. A small but significant boost of α-SMA–positive cells driven by microRNA mimics was observed in vitro (microRNA-27b-5p mimic, *P*=0.013; microRNA-497-5p mimic, *P*=0.005 versus scramble; Mann-Whitney *U* test; Figure 4Ai through 4Av and 4B). This effect was accompanied by the expected regulation of the relevant microRNA expression in both gain-of-function and loss-of-function experiments (Figure 4C). Unlike our previous experiment conducted in healthy rCFs, transfection of failing rCFs with microRNA inhibitors decreased expression of α-SMA only (Figure 4D and 4E; Figure S4Bii and S4Cii).

### MicroRNA-27b-5p and MicroRNA-497-5p Regulate Hypoxia-Inducible Factor 1 Signaling by Targeting Egl-9 Family Hypoxia-Inducible Factor

**Figure 5.**
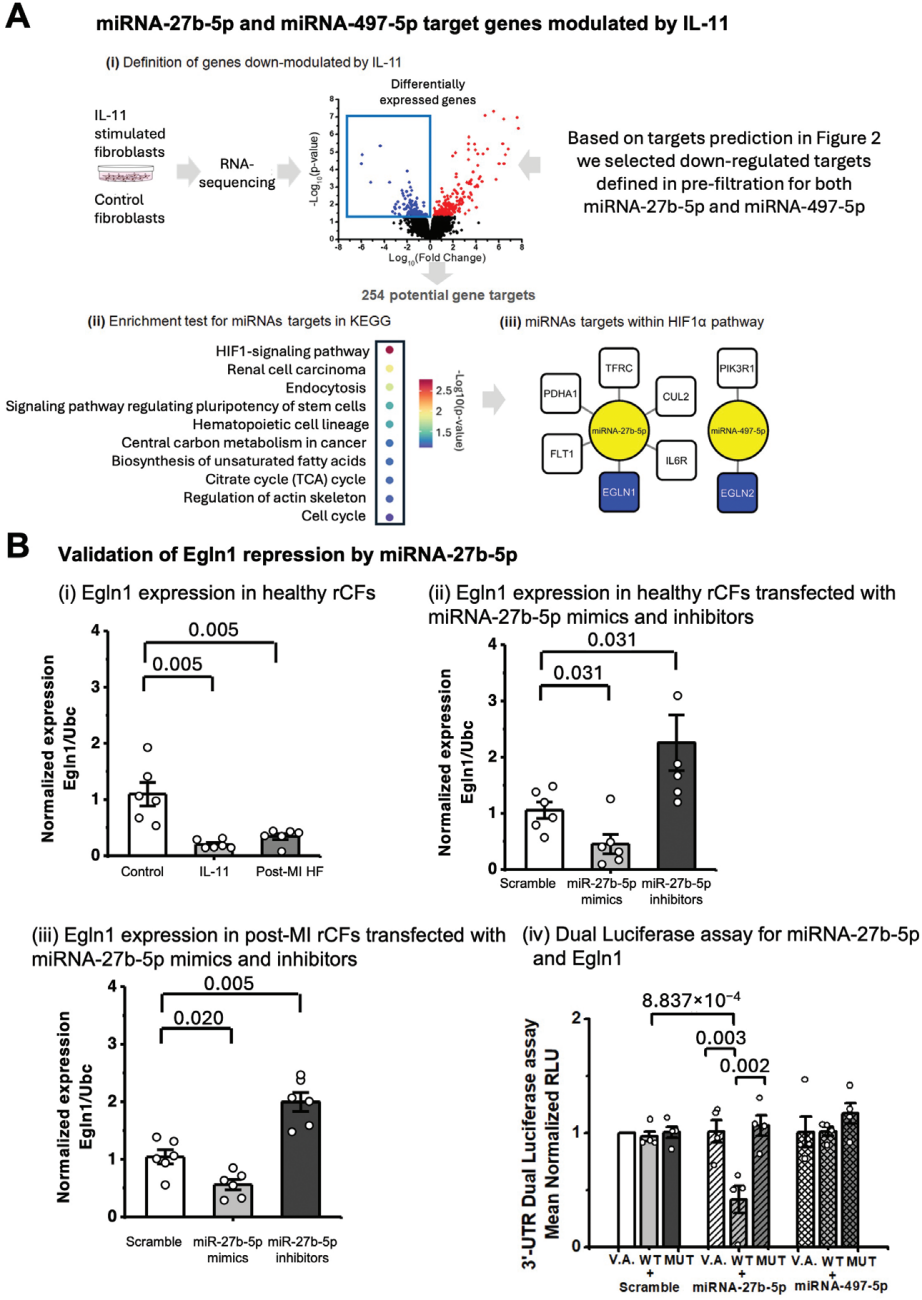
**Identification, prioritization, and validation of EGLN1 (Egl-9 family hypoxia-inducible factor 1) as microRNA (miRNA)-27b-5p target. A**, Schematic description of the target prediction process by using (**i**) downregulated genes in human cardiac fibroblasts (hCFs) stimulated with IL (interleukin)-11. **ii**, Enrichment test: graph is plotted as −log10(*P* values), with *P*<0.05. **iii**, miRNA targets network from HIF (hypoxia-inducible factor)-1 signaling pathway; miRNAs—yellow, EGLN1, 2—blue, remaining targets—white. Among the remaining targets: FLT1 (Fms related receptor tyrosine kinase 1), PDHA1 (pyruvate dehydrogenase E1 subunit alpha 1), TFRC (transferrin receptor), CUL2 (cullin 2), IL6R (interleukin 6 receptor), PIK3R (phosphatidylinositol 3-kinase regulatory subunit alpha). Bar charts display reverse transcription quantitative PCR (RT-qPCR) analysis of EGLN1 expression normalized to UBC (ubiquitin C) (N=6, n=3). **B**, Transfection of healthy rat cardiac fibroblasts (rCFs) (**ii**) or post–myocardial infarction heart failure rCFs (**iii**) with scramble nucleotides (white), miRNA-27b-5p or miRNA-497-5p mimic (light gray), and inhibitor (dark gray) at 5 nmol/L concentration. **Bi**, rCFs activated by IL-11 (5 ng/mL, 24 hours) and rCFs from post-MI HF rats. **Biv**, 3′-untranslated region (UTR) dual luciferase assay in human embryonic kidney (HEK) 293FT cells with a combination of vector alone (V.A.), wild-type miRNA-27b-5p binding site (wild-type [WT]), or a mutated miRNA-27b5p binding site (MUT) for EGLN, cotransfected with a plasmid encoding Renilla luciferase along with a plasmid encoding either miRNA-27b-5p; scramble sequence (scr), or miRNA-497-5p. Firefly luciferase luminescence was normalized against Renilla luminescence (presented as relative luminescence units [RLU]). Experimental values were compared with the vector-only and scramble vector transfection conditions. The Mann-Whitney *U* test was used to determine statistical significance in **Bi** through **Biii**. One-way ANOVA was used for **iv**, *P* values shown. KEGG indicates Kyoto Encyclopedia of Genes and Genomes; and TCA, tricarboxylic acid cycle.

We predicted 596 and 140 potential gene targets of microRNA-27b-5p and microRNA-497-5p, respectively. These were shortlisted to 254 predicted targets differentially expressed in hCFs stimulated with IL-11 (Figure 5Ai). This refined list was used to perform pathway enrichment analysis (Figure 5Aii), which revealed that the HIF (hypoxia-inducible factor) 1 signaling pathway contained many of the potential targets of microRNA-27b-5p and microRNA-497-5p. Both microRNAs (Figure 5Aiii, in yellow) were predicted to target the EGLN (Egl-9 family hypoxia-inducible factor) gene family (Figure 5Aiii, in blue). The EGLN genes encode PHD (prolyl hydroxylase domain) enzymes, which target HIF-α signaling by promoting its polyubiquitination and proteasomal degradation.^26^ EGLN plays a critical role in regulating HIF-α abundance and oxygen homeostasis.^26^ Increased EGLN1 (RNA)/PHD2 (protein) was recently shown to be an important player in cardiac fibrosis and hypertrophy.^27^ MicroRNA-497-5p targeting of EGLN2 has already been demonstrated.^28^ We, therefore, embarked on validating the direct binding and expressional regulation of EGLN1 by microRNA-27b-5p.

Egln1 was significantly downregulated in IL-11-treated rCFs and in failing rCFs compared with healthy rCFs (*P*=0.005—both comparisons; Mann-Whitney *U* test; Figure 5 Bi). In both healthy (Figure 5Bii) and pathological failing CFs (Figure 5Biii), microRNA-27b-5p loss-of-function increased Egln1 expression. In failing rCFs, microRNA-27b-5p overexpression resulted in Egln1 downregulation. To further evaluate the functional role of the microRNA-27b target in regulating IL-11–induced cardiac fibrosis, we operated a dual approach in IL-11–stimulated CF: (1) we tested the impact of microRNA-27b-5p inhibitor on Egln1 mRNA expression (Figure S6A) and (2) we overexpressed Egln1 (Figure S6B). After IL-11 stimulation, Egln1 mRNA expression levels were significantly rescued in cells pretreated with the microRNA-27b-5p inhibitor compared with those pretreated with scramble oligonucleotides (*P*=0.019; ANOVA with Tukey post hoc test; Figure S6A). Moreover, Egln1 overexpression in CF attenuated the IL-11–induced mRNA expression of myofibroblast markers αSMA and Postn (Figure S6C). This profibrotic role of microRNA-27b-5p was also observed in human CFs (Figure S7), alongside a decreased expression of EGLN1, and upregulation of HIF2α (Figure S7Dii and S7Diii).

Next, to validate the EGLN1 direct targeting by microRNA-27b-5p, we conducted a dual luciferase assay (Figure 5Biv). Our results demonstrated a significant decrease in relative luminescence when microRNA-27b-5p was cotransfected with the pMIR luciferase reporter containing the wild-type EGLN1 binding site (*P*=0.003; ANOVA with Tukey post hoc test), but not in the presence of the mutated binding site (MUT EGLN1).

### MicroRNA-497-5p and MicroRNA-27b-5p Are Upregulated in the Heart of Tg-Il11 Mice

To evaluate the role of microRNA-27b-5p and microRNA-497-5p in vivo, we assessed their expression levels in the LV of tamoxifen-inducible transgenic mice (Tg-Il11), allowing a cardiomyocyte-specific *Il11* overexpression (Figure S8A and S8B).^29^ In comparison with controls, LV tissues of Tg-Il11 mice exhibited higher Il11 RNA expression (Figure S8C) along with profibrotic markers, such as Col1a1, α-SMA, Mmp2, and Tgfβ1 (Figure S8F, S8Hi through S8Hiii). Importantly, microRNA-27b-5p and microRNA-497-5p expression levels were increased (Figure S8Di and S8Dii), whereas their predicted hypoxia-related targets Egln1 and Egln2 were decreased in the Tg-Il11 mice (Figure S8GI and S8Gii). Conversely, HIF-1α mRNA—which encodes a protein targeted for degradation by Eglns—was upregulated in Tg-Il11 mice (Figure S8Giii). Both microRNAs were positively correlated with Il11 expression in LV tissues of Tg-Il11 mice and littermate controls (microRNA-27b-5p: *r*=0.47, *P*=0.03; microRNA-497-5p: *r*=0.41, *P*=0.03; Pearson correlation; Figure S8EI and S8Eii).

### MicroRNA-27b-5p and MicroRNA-497-5p Are Upregulated in the LV of TAC Mice

To further investigate the role of microRNA-27b-5p and microRNA-497-5p in vivo, we generated a TAC mouse model.^30^ Measurement of the peak gradient at the constriction site on the aortic arch at day 7 showed an increase in the TAC-operated animals (Figure S9A). Transthoracic echocardiography performed 60 days postsurgery revealed a decrease in fractional shortening and LV ejection fraction (LVEF) and an increase in LV mass (Figure S9B and S9C). In addition, TAC resulted in an increase of LV volume and LV internal diameter during both systole and diastole (Figure S9DI, S9Dii, S9Fi, and S9Fii).

Expression of profibrotic markers, including Col1a1, Mmp2, and Ctgf (Figure S10G and S10F), was elevated in LV tissues 60 days postsurgery. MicroRNA-27b-5p and microRNA-497-5p also showed a significant upregulation in the TAC mice (*P*=0.014 and *P*=0.025; Mann-Whitney *U* test; Figure S10Bi and S10Bii), along with increased Il11 expression (Figure S10A). Both microRNAs were positively correlated with Il11 expression in LV tissues of TAC mice and sham-operated controls (*r*=0.77, *P*=0.0001–microRNA-27b-5p; r=0.64, *P*=0.005–microRNA-497-5p; Pearson correlation; Figure S10Ci and S10Cii). Furthermore, Egln1 and Egln2 were downregulated in the TAC mice (Figure S10Ei and S10Eii).

### Tissue Expression of MicroRNA-27b-5p and MicroRNA-497-5p in Patients With AS

**Figure 6.**
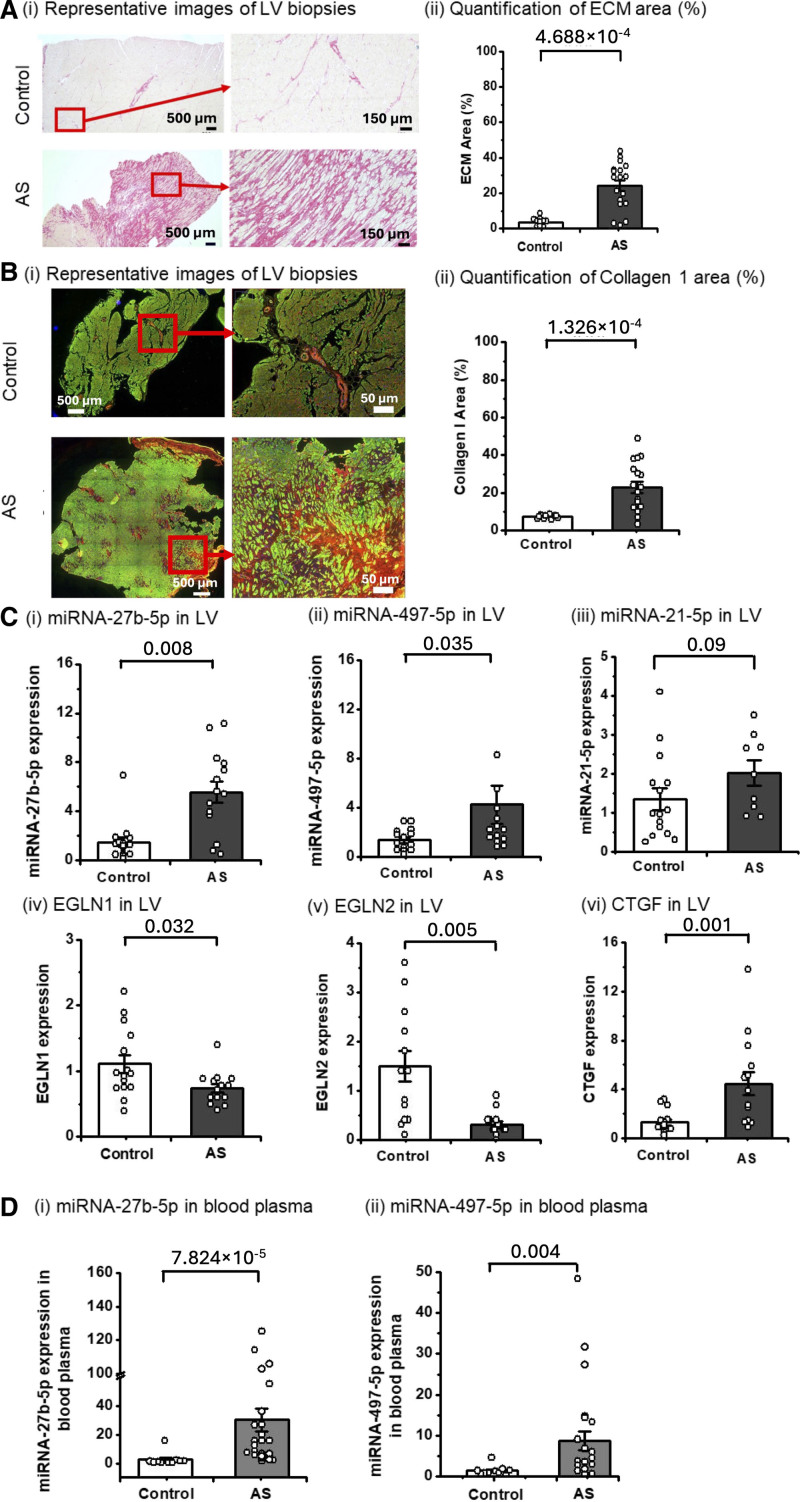
**MicroRNA (miRNA)-27b-5p and miRNA-497-5p are upregulated in left ventricular (LV) and plasma of patients with aortic stenosis (AS), characterized by increased collagen deposition. A**, Histological analysis of LV biopsies from a control and AS groups (**i**) representative images of LV biopsies stained with Sirius red from the control group and patients with AS. **ii**, Quantification of ECM (extracellular matrix) area (%) in LV biopsies stained with Sirius red in control (white; N=10) and AS group (dark gray; N=17). **B**, Collagen I staining LV biopsies from a control and AS groups (**i**) representative images of LV biopsies collagen I (red), α-sarcomeric actin (green), and DAPI (4′,6-diamidino-2-phenylindole; blue); (**ii**) quantification of collagen I area. **C**, Gene expression normalized to UBC (ubiquitin C) for mRNA and to U6 for miRNAs in LV. Gene expression in LV of AS (dark gray) N=14 and control (white) patient N=14 of (**i**) miRNA-27b-5p, (**ii**) miRNA-497-5p, (**iii**) miRNA-21–5p (N=15–control, N=9–AS; **iv**) EGLN1 (Egl-9 family hypoxia-inducible factor 1), (**v**) EGLN2, (**vi**) CTGF (connective tissue growth factor). **D**, miRNA expression in plasma was normalized to preadded spike-in (cel-miR-39-5p or caenorhabditis elegans microRNA-39-5p). miRNAs expression: (**i**) miRNA-27b-5p, (**ii**) miRNA-497-5p in plasma of AS (gray) N=24 referred to a control group (N=10). The Mann-Whitney *U* test was performed to estimate statistical significance, and *P* values are shown.

LV samples were collected from patients with AS undergoing concomitant septal myectomy during surgical aortic valve replacement (N=29) and healthy donors (N=35, Table S1). AS samples exhibited extensive replacement and interstitial fibrosis when compared with the control group, quantification of the sirius red staining demonstrated a significant increase of ECM area in patients with AS (*P*=4.688×10^−4^; Mann-Whitney *U* test; Figure 6A). Quantification of collagen I area in these biopsies revealed significantly higher levels of fibrosis in patients with AS (*P*=1.326×10^−4^; Mann-Whitney *U* test; Figure 6B).^31^ We also observed significant upregulation of CTGF (connective tissue growth factor) in LV of patients with AS (*P*=0.001; Mann-Whitney *U* test; Figure 6Cvi), indicating myocyte injury.^32^

The expression of microRNA-27b-5p and microRNA-497-5p was elevated in the LV of patients with AS (Figure 6Ci and 6Cii). In keeping with our proposed pathway, the microRNA-regulated targets EGLN1 and EGLN2 had reduced expression in the same biopsies (Figure 6Civ and 6Cv). There were no significant differences in the concentrations of either microRNA in LV of patients with AS when subdivided by age, hypertension, or dyslipidemia (Figure S11). In addition, the expression level of microRNA-21-5p in AS discovery cohort patients LV biopsies was comparable to donor biopsies (Figure 6Ciii).

### Plasma MicroRNA-27b-5p and MicroRNA-497-5p as Indicators of Cardiac Fibrosis

**Figure 7.**
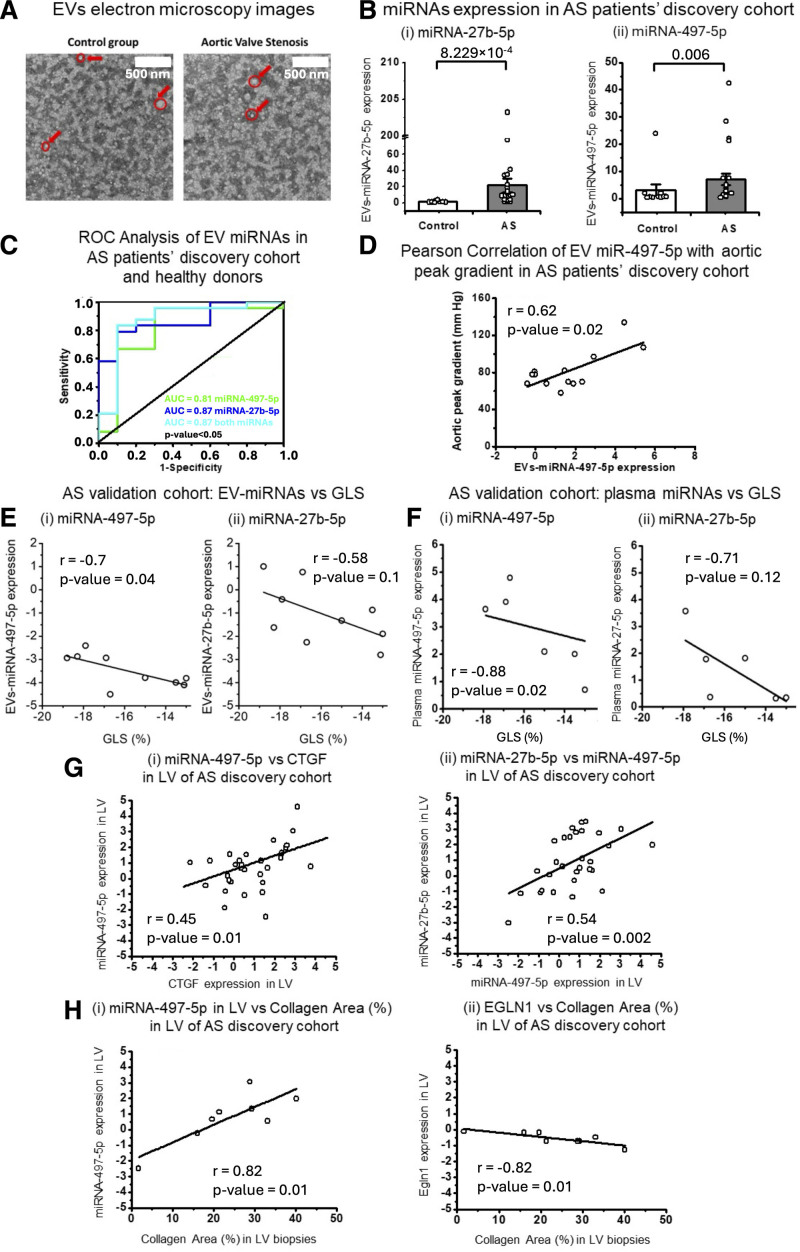
**MicroRNA (miRNA)-27b-5p and miRNA-497-5p expression in extracellular vesicles (EVs) from patient plasma is correlated with fibrotic markers. A**, Representative images of EVs isolated from plasma. Red arrows and circles highlight single EVs. **B**, Expression of (**i**) miRNA-27b-5p and (**ii**) miRNA-497-5p in EVs from aortic stenosis (AS) discovery cohort plasma (N=23) and donors (N=10). Statistical significance was evaluated with Mann-Whitney *U* test, and *P* values are shown. **C**, Receiver operating characteristic (ROC) curve analysis based on expression of miRNA-497-5p (green), miRNA-27b-5p (blue), both (cyan) in EVs from plasma of AS discovery cohort (N=24) and healthy donors (N=10). Area under the curve is measured and presented on the graph along with *P* values. **D**, Pearson correlation of normalized expression of miRNA-497-5p in EVs from plasma of AS discovery cohort patients with patients’ aortic peak gradient (mm Hg; N=14). **E** and **F**, Pearson correlation analysis of miRNA-497-5p (**Fi** and **Fii**) and miRNA-27b-5p (**Ei** and **Eii**) expression in plasma (**F**) or plasma-derived EVs (**E**) from AS validation cohort patients (N=6 for plasma, N=9 for EVs), with values normalized to the preadded spike-in (cel-miR-39-5p)**. G**, Pearson correlations on measured parameters in AS discovery cohort patients between (**i**) normalized expression of miRNA-497-5p in left ventricular (LV) with normalized expression of CTGF (connective tissue growth factor) in LV (N=29); (**ii**) normalized expression of miRNA-27b-5p in LV with normalized expression of miRNA-497-5p in LV (N=29). **H**, Pearson correlations on measured parameters in AS discovery cohort patients between (**i**) normalized expression of miRNA-497-5p in LV of patients with AS with collagen area (%) measured in LV biopsies stained with Sirius red (N=8); (**ii**) mean normalized expression of EGLN1 (Egl-9 family hypoxia-inducible factor 1) in LV with collagen area (%) measured in LV stained with Sirius red (N=8). AUC indicates area under the curve; and GLS, global longitudinal strain.

We measured the expression of both microRNA-27b-5p and microRNA-497-5p in the peripheral blood plasma of discovery cohort patients with AS, comparing their expression with sex matched healthy donors. The levels of both microRNAs were increased in plasma of patients with AS (Figure 6Di and 6Dii). There was no involvement of the confounding factors: age, hypertension, and dyslipidemia (Figure S12). Global longitudinal strain (GLS), derived from contemporaneous patient echocardiograms, was assessed in patients from the AS validation cohort (N=14; Tables S2 and S3). Plasma levels of microRNA-27b-5p and microRNA-497-5p demonstrated a negative correlation with GLS (Figure 7Fi and 7Fii). Suggesting that these circulating microRNAs may serve as indicators of early fibrotic changes and preserved ventricular mechanics.

### Evaluation of EV-Associated MicroRNA-27b-5p and MicroRNA-497-5p as Circulating Biomarkers for Cardiac Fibrosis

MicroRNAs can circulate in the blood encapsulated in EVs.^20,33^ We isolated EVs from an AS patient (discovery and validation cohorts) and healthy donor plasma. EVs (50–130 nm diameter) were characterized by transmission electron microscopy with negative uranyl acetate staining (Figure 7A). To investigate the compartmentalization of microRNA-27b-5p and microRNA-497-5p in plasma, we treated healthy donor plasma samples with proteinase K to disrupt ribonucleoprotein complexes such as AGO2 (argonaute-2).^34^ MicroRNAs normally bound in such complexes are released and become susceptible to RNase-mediated degradation. The positive (microRNA-16-5p) and negative (let-7a-5p) controls confirmed that proteinase K treatment of plasma was effective (Figure S13Ai and S13Aii). MicroRNA-27b-5p detection did not change after treatment, indicating location within EVs (Figure S13Bi). MiRNA-497-5p detection was decreased after the proteinase K treatment, albeit to a lesser extent than microRNA-16-5p, suggesting that microRNA-497-5p may be present in both vesicles and ribonucleoprotein complexes (Figure S13Bii).

MicroRNA-27b-5p and microRNA-497-5p levels were significantly higher in EVs derived from the AS patient discovery cohort (*P*=8.229×10^−4^ and *P*=0.006; Mann-Whitney *U* test; Figure 7B). Receiver operating characteristic (ROC) curves were plotted based on microRNA levels in EVs isolated from plasma (Figure 7C). EV-associated microRNA-27b-5p and microRNA-497-5p discriminated patients with AS from controls in a sensitive and specific manner (area under the curve=0.81–microRNA-497-5p; area under the curve=0.87–microRNA-27b-5p). The combination of both microRNAs did not further increase sensitivity and specificity (area under the curve=0.87). We found that microRNA-497-5p expression in LV positively correlated (*r*=0.82, *P*=0.01; Pearson correlation) with collagen area (%; Figure 7Hi), CTGF expression (*r*=0.45, *P*=0.01; Pearson correlation; Figure 7Gi) and with microRNA-27b-5p expression in LV (*r*=0.54, *P*=0.002; Pearson correlation; Figure 7Gii). At the same time, EGLN1, was negatively correlated (*r*=−0.82, *P*=0.01) with collagen area (%; Figure 7Hii). MicroRNA-497-5p levels in EVs derived from plasma of patients with AS were positively correlated (*r*=0.62, *P*=0.02; Pearson correlation) with aortic peak gradient in patients with AS (Figure 7D).

In addition, we tested how risk factors such as age, hypertension, and dyslipidemia affected microRNA-27b-5p and microRNA-497-5p expression in plasma and EVs derived from plasma (Figure S12). We found that these factors did not affect microRNA expression. However, microRNA-497-5p expression in EVs derived from plasma demonstrated some dependence on patient age (Figure S12L). Consistent with data obtained using our AS discovery cohort, we found that EV-derived microRNA-27b-5p and microRNA-497-5p expression was negatively associated with GLS in the AS validation cohort (Figure 7E).

## Discussion

Fibrosis represents a key feature of myocardial damage induced by AS and marks the transition between the compensatory hypertrophy with interstitial fibrosis and HF associated with replacement fibrosis.^35^ The results of our study indicate microRNA-27b-5p and microRNA-497-5p are IL-11-responsive mediators of cardiac fibrosis. Moreover, we have identified the potential of the 2 microRNAs as circulating biomarkers of cardiac fibrosis in patients with AS with preserved ejection fraction.

We report that the profibrotic IL-11 increases microRNA-27b-5p and microRNA-497-5p in vitro in healthy rat CF, and in vivo in LV tissues of Tg-Il11 mice. Moreover, the 2 microRNAs are upregulated in the heart of TAC-operated mice as well as in CFs derived from post-MI HF rats.

MicroRNA-27b-5p and microRNA-497-5p were previously investigated in the context of fibrosis.^36–38^ MicroRNA-27b enhances cardiac fibrosis,^36^ whereas microRNA-497-5p regulates ECM remodeling in the lungs and liver.^37,38^ MicroRNA-27b was also shown to be relevant for cardiovascular disease—its transgenic overexpression in vivo promoted hypertrophy, whereas microRNA-27b silencing in TAC mice attenuated cardiac hypertrophy and dysfunction.^39^

Our data also highlights a potential mechanism of fibrosis induction by microRNAs through expressional repression of EGLN. EGLN loss has been functionally implicated with cardiac fibrosis and hypertrophy.^27,40^ Recently, Dai et al^27^ demonstrated that mice with endothelial-specific *EGLN1* gene deletion exhibited LV hypertrophy and cardiac fibrosis, and identified HIF2α as a transcriptional factor responsible for the profibrotic signaling. Interestingly, IL-11 differential coexpression network is enriched in genes associated with hypoxia (eg, *HIF2α*, *VEGFC*, *CITED2*).^41^ These findings are in keeping with previous reports of EGLN2 as a target of microRNA-497-5p,^28^ and with our demonstration that microRNA-27b-5p directly binds to and suppresses EGLN1 mRNA expression. Reduced Egln1 expression was also observed in rat CFs treated with IL-11 and rat CFs extracted from the post-MI failing hearts. Moreover, LV Egln1 and Egln2 mRNA levels were reduced in our 2 mouse models of cardiac fibrosis, and remarkably, also in the LV of patients with AS. IL-11–induced microRNA-27b-5p and microRNA-497-5p may therefore drive an excessive and profibrogenic hypoxia signaling in a clinically relevant setting via regulating both ELGN1 and ELGN2.

The use of circulating biomarkers has been encouraged in recent guidelines for AS monitoring and management.^42^ There is a clear correlation between BNP (B-type natriuretic peptide) active levels and mortality, even in asymptomatic patients with AS.^43^ However, a biomarker analysis of the EARLY TAVR trial, recently showed that NT-proBNP (N-terminal pro-B-type natriuretic peptide) and high-sensitivity troponin are unable to identify patients who would benefit from an early treatment, limiting their clinical value.^44^ Specific biomarkers associated with adverse fibrotic remodeling of the myocardial tissue in AS could guide the necessity and timing of clinical interventions more precisely.

Recent studies have highlighted the involvement of microRNAs in the progression of cardiac fibrosis through their modulation of fibrosis-related genes and signaling pathways.^45^ In our hands microRNA-27-5p and microRNA-497-5p hold potential as biomarkers of early myocardial fibrosis. Current ESC/EACTS guidelines recommend initiating treatment of AS when patients begin to experience symptoms or in case of LV impairment.^42^ However, symptoms are difficult to assess in patients with multimorbidity, whose survival rates decrease dramatically after the onset of symptoms.^46^ In asymptomatic patients, treatment is recommended for individuals with preserved LV function and critical or rapidly progressing AS or when LVEF <55% without another cause. Historically, the cutoff for LV dysfunction has been considered LVEF <50%. In a large retrospective multicentric study including 1678 patients with severe AS and preserved LV function, LV function below 55% was associated with poor outcomes, despite treatment,^24^ emphasizing the importance of early recognition of myocardial damage. LV GLS could be a potential surrogate for assessing early cardiac damage. A meta-analysis including 1067 asymptomatic patients with severe AS showed that impaired LV GLS was associated with reduced survival.^47^ These results were further confirmed in asymptomatic patients with an updated echocardiographic scoring system in which mild LV dysfunction (LVEF <60%) was associated with increased mortality.^23^ These data indicate that myocardial function, assessed by echocardiogram, may not be able to identify early myocardial damage in AS.

We observed an increase in the expression of circulating microRNA-27-5p and microRNA-497-5p in patients with AS with preserved ejection fraction. We demonstrated that in patients with AS with preserved ejection fraction (LVEF >55%) and intensive myocardial ECM accumulation, both microRNA-27b-5p and microRNA-497-5p are upregulated in the LV, plasma, and plasma-derived EVs. Correlation analysis revealed a strong correlation of microRNA-497-5p levels in LV with CTGF, which plays a crucial role in fibrosis development and progression,^32^ as well as with collagen area in histological sections. In our prospectively collected validation cohort of patients with AS, we additionally observe a negative correlation between circulating microRNA-27b-5p and microRNA-497-5p expression levels with GLS. Our data suggest that microRNA-497-5p demonstrates greater sensitivity to fibrosis-associated parameters compared with microRNA-27b-5p. To further investigate this observation, we analyzed the expression patterns of their primary transcripts (pri-microRNAs) across diverse tissues and cell types using 2 complementary computational resources: the PPMS framework for profiling pri-microRNAs from single-cell RNA-sequencing data,^48^ and the microRNA Tissue Atlas.^49^ Both analyses confirmed the multiorgan and multicellular expression of pri-miR-497 and pri-miR-27b, indicating that neither microRNA is restricted to a specific tissue or cell type. Notably, we observed that microRNA-497-5p is endogenously expressed at lower levels than microRNA-27b-5p in human LV and blood. This lower baseline expression may partially explain its enhanced sensitivity to fibrosis-related changes. Although we did not observe significant differences in sensitivity between plasma-derived and EV-derived microRNAs, we think these findings have strong clinical relevance as blood samples only require standard processing before biomarker detection.

We propose that microRNA-27b-5p and microRNA-497-5p could be useful biomarkers for: (1) risk stratification of patients with symptomatic severe AS and (2) to determine the timing of clinical intervention for asymptomatic patients with severe AS with preserved LV.

### Clinical Perspectives

Several studies have demonstrated the importance of the identification of myocardial fibrosis on CMR. In particular, the presence of focal scar detected with LGE and its extent were significantly correlated with mortality.^23,31,35^ CMR remains an expensive, time-consuming resource, not available in all centers, and not suitable for all patients with AS.^24^ For these reasons, the identification of circulating biomarkers of fibrosis associated with AS, could provide a cost- and time-effective solution to assess fibrosis. Recently, the EVoLVeD trial (Early Valve Replacement Guided by Biomarkers of LV Decompensation in Asymptomatic Patients With Severe AS) failed to demonstrate a benefit of early intervention in patients with asymptomatic severe AS with mid-wall LGE and subclinical decompensation in the composite outcome of mortality and unplanned aortic-related hospitalization.^50^ However, the patients included in this trial had documented mid-wall replacement fibrosis on CMR and increased circulating troponin I levels, which might represent a late stage in the spectrum of AS-related LV damage, and many of the reported deaths were not related to the AS.

There is a clear clinical need for markers of early myocardial injury and remodeling to guide timely intervention. The EARLY TAVR and EVoLVeD trials demonstrated that the current guideline biomarkers, high-sensitivity troponin and BNP, fail to identify patients who could benefit from early intervention and hospitalization.^44,50^ This underscores the potential value of novel biomarkers such as the microRNAs investigated in our study. The extremely selected population included in our study, which excludes the other possible cardiac and noncardiac causes of LV fibrosis, highly correlates with our clinical findings of LV fibrosis secondary to AS. This could limit the study of microRNA-27-5p and microRNA-497-5p to a highly selected population. Future studies are needed to validate the study of these microRNAs in a larger cohort of patients with concomitant comorbidities to assess specificity.

### Translational Outlook

We assert that fibrosis-promoting microRNA-27-5p and microRNA-497-5p hold potential as both biomarkers of early myocardial fibrosis. We have demonstrated that both microRNAs are critical components of the IL-11 profibrotic mechanism. In particular, we have newly identified a profibrotic pathway initiated by IL-11 in CFs: EGLN1 suppression by microRNA-27b-5p. The use of these molecules as circulating biomarkers could assist clinicians with risk stratification of patients with a diagnosis of severe AS, allowing them to prioritize interventions in patients with increased levels of cardiac fibrosis. The prognostic implications of these microRNAs would be complementary to the current practice, providing an objective, measurable, and reliable indicator of cardiac damage in a population where symptoms are difficult to assess. Moreover, understanding the pathway involved in cardiac fibrosis in AS could open considerations for new targets for therapy, which could be complementary to the treatment of AS, protecting the myocardium once the diagnosis of AS has been made.

## Article Information

### Acknowledgments

The authors are grateful to Maria Carla Panzeri and Valeria Berno (San Raffaele Hospital) and the other staff of the Advanced Light and Electron Microscopy BioImaging Center (ALEMBIC) in San Raffaele Hospital for the technical assistance. The authors are thankful to Marialucia Longo (Istituto di Ricovero e Cura a Carattere Scientifico [IRCCS] Policlinico San Donato) for providing human heart tissue sections. The authors thank the Cellular Mechanosensing and Functional Microscopy Centre of Excellence at Imperial College London.

### Disclosures

None

### Supplemental Material

Supplemental Methods

Tables S1–S5

Figures S1–S13

Major Resources Table

References 51–67

## Supplementary Material
