# Supplementary material for: Interleukin 11–Induced MicroRNAs as Functional Mediators and Circulating Biomarkers of Cardiac Fibrosis
Source: Circ Res. 2026 Mar 17;138(9):e326799. doi: 10.1161/CIRCRESAHA.125.326799 (PMC13108434; doi:10.1161/CIRCRESAHA.125.326799)
Supplement: Supplementary file 1 [file res-138-e326799-s001.docx]

1. **Supplemental Material: Interleukin 11-induced microRNAs as functional**
2. **mediators and circulating biomarkers of cardiac fibrosis**

**Supplemental methods**

## Transverse aortic constriction mouse model

Male *C57Bl/6N* mice, aged two months, were obtained from Charles River Laboratories (Calco, Italy). Only male mice were used, which reflected the need for protocol harmonization and reduced biological variability across multiple institutions during initial mechanistic studies.^51^ We recognize this as a limitation, as sex influences cardiac fibrosis and remodeling after transverse aortic constriction (TAC).^51^ All animals were housed under standard specific pathogen-free conditions with a 12 h light-dark cycle, controlled temperature (22-23^o^C) and humidity (45-50%). Mice were fed a standard laboratory chow diet (VRF1 (P), Special Diet Services, 801900) and had ad libitum access to food and water. Animals were randomly assigned to either the control (sham) or TAC group prior to surgery using a simple randomization ‘numbers-out-of-a-hat’ method. Animal numbers were determined a priori from power calculations with 80% power and a 95% confidence interval using standard deviation values from our previously published work.^52^ Mice underwent sham or TAC surgery under ketamine (100 mg/kg, Ketavet 100; Intervet Farmaceutici) and medetomidine (0.5 mg/kg, Domitor; Orion Pharma) anesthesia, following established protocols.^30,52^ A bent 27-gauge needle was used to constrict the aorta between the brachiocephalic and left carotid arteries, and atipamezole (5 mg/kg, Antisedan; Orion Pharma) was administered post-surgery to aid recovery. Sham mice underwent identical procedures without aortic ligation.

Ultrasound imaging was performed using a high-performance ultrasonographic imaging system (Vevo 2100; Fujifilm VisualSonics Inc., Toronto, ON, Canada). The aortic arch view was obtained from a modified right parasternal view on day 7 after surgery. Transthoracic echocardiography was performed at 60 days after surgery. Two-dimensional short-axis images and M-mode recordings were observed at the level of the papillary muscles. From the M-mode recordings, anatomical parameters in diastole and systole were obtained by Vevo Lab 3.2.6 software (Fujifilm VisualSonics Inc., Toronto, ON, Canada). The echocardiographic parameters obtained in sham-operated mice on day 60 were used as reference values.

Mice were sacrificed at 60 days post-TAC surgery and samples were snap-frozen in liquid nitrogen and stored at -80 °C until use.

## Tg-Il11 mouse model

As previously described, the cardiomyocyte-specific *Il11* transgenic mouse model (Tg-Il11) was generated using the Cre-loxP system.^29^ Briefly, *Rosa26-Il11* mice, carrying a *loxP-*flanked STOP cassette upstream of the *Il11* transgene under a pCAG promoter, were crossed with *α-MHC-MerCreMer* mice to enable tamoxifen-inducible Cre expression in cardiomyocytes. Half of the offspring (Tg-Il11 or *MCM^Cre/+^ R26R^Il11/+^*) exhibited *Il11* overexpression, while littermates (*MCM^Cre/+^ R26R^+/+^*) served as controls. Both male and female animals were included in the study. Animal numbers were determined a priori from power calculations with 80% power and a 95% confidence interval using standard deviation values from our previously published work.^29^ All animals were housed under standard specific pathogen-free conditions with a 12 h light-dark cycle, controlled temperature (22-23^o^C) and humidity (45-50%). Mice were fed a standard laboratory chow diet (RM1, Special Diet Services, 1030) and had ad libitum access to food and water throughout the study. Tamoxifen (Sigma-Aldrich, MO, USA, T5648) was dissolved in ethanol (Fisher Scientific, MA, USA, 10437341, 10% v/v) and corn oil (Sigma-Aldrich, C8267) at 55 °C and protected from light. Tamoxifen was administered intraperitoneally from 8 weeks of age for 5 consecutive days at a dose 20 mg/kg. Mice were sacrificed 5 weeks after transgene induction and samples were snap frozen in liquid nitrogen and stored at -80^o^C until use.

## Myocardial infarction rat model

Rats were housed under standard specific pathogen-free conditions with a 12 h light-dark cycle, controlled temperature (22-23 °C) and humidity (45-50%). Rats were fed a standard laboratory chow diet (T2014X, [Teklad Global Diets^®^](https://www.inotiv.com/hubfs/resources/brochures/teklad-global-diets-product-line.pdf)) and had ad libitum access to food and water throughout the study. Only male rats were used, which reflected the need for protocol harmonization and reduced biological variability across multiple institutions during initial mechanistic studies across multiple institutions.^53^ We recognize this as a limitation, as sex influences cardiac fibrosis and remodeling in established myocardial infarction (MI) models.^53^ Animals were randomly assigned to the MI or control group prior to surgery using a simple randomization ‘numbers-out-of-a-hat’ method. Animal numbers were determined a priori from our previously published works.^41,54^ MI was performed by proximal coronary ligation as previously described.^54^ In brief, coronary ligation was performed on male Sprague Dawley rats to generate transmural infarcts and induce chronic heart failure. 16-24 weeks postinfarction, the infarcted heart significantly increased in heart weight/body weight ratios (g/kg) and the heart showed significantly reduced ejection fraction and shortening (p =0.031; Mann-Whitney U test) (Figure S1 A-C) and elevated end-diastolic pressure, suggested hypertrophy of the heart as well as decreased cardiac output and function. Animals that failed to reach 16 weeks post-infarction due to surgery complications were excluded from the study.

## Cell culture of human and rat fibroblasts

Human and rat cardiac fibroblasts (hCFs and rCFs) were isolated by enzymatic digestion of ventricular tissue as previously described.^41^

rCFs were isolated from the LV of animals that were either healthy, sham-operated, or at 16 weeks post-MI and have developed HF, as confirmed by echocardiography-ascertained reduction of LV fractional shortening and ejection fraction (Figure S1).

Both hCFs and rCFs were cultured in Dulbecco's modified Eagle's media (DMEM, D0819) supplemented with 10% fetal bovine serum (FBS, F9665) and 1% antibiotic-antimycotic solution (A5955). Cells were maintained at 37 °C with 5% CO_2_. Isolation and culturing protocols resulted in high purity of fibroblasts, confirmed by PDGFRα and vimentin staining (Figure S2). In the experimental conditions, vimentin was used as a cytosolic stain of fibroblasts and myofibroblasts, while α-SMA was used as a myofibroblast-specific marker. Both hCFs and rCFs were cultured for no longer than 3 weeks (passages 3-5). Cells were seeded before experiments in a 12-well plate at 70% confluence.^55^

**Cardiac fibroblasts treatment and transfection**

At 24 hours after seeding, rat cardiac fibroblasts (rCFs) were treated with IL-11 (5 ng/mL) or an equal volume of PBS for 24 hours.

For the miRNA-27b-5p and miRNA-497-5p gain- and loss-of-function experiments, rCFs were plated for 24 hours and subsequently transfected with following oligonucleotides: universal scramble control nucleotides (scr) (Horizon Discovery, D-001810-10-20), or with mirVana^TM^ mimics/inhibitors: mmu-miR-497a-5p mimic (mim) (Cat. Number MC11293), mmu-miR497a-5p inhibitor (inh) (Cat. Number MH11293), rno-miR-27b-5p mimic (Cat. Number MC20018) and rno-miR-27b-5p inhibitor (Cat. Number MH20018). mirVana^TM^ mimics for miRNA-497-5p were designed to work in both rat (rno-) and mouse (mmu-) model. All oligonucleotides used for this study were utilized at a concentration of 5 nM. Transfection was performed with Lipofectamine™ 3000 Transfection Reagent (Thermo Fisher Scientific, L3000001) according to the manufacturer's protocol.

For the miRNA-27b-5p gain and loss of function experiments hCFs were seeded as described previously for 24 hr. As a further step, cells were transfected with following oligonucleotides: scramble nucleotides (scr) (Horizon Discovery D-001810-10-20), or mirVana mimics and inhibitors: hsa-miR-27b-5p (Cat. Number MC12831, miRVana) and hsa-miR-27b-5p (Cat. Number MH12831, miRVana) respectively. Oligonucleotides used for this study were utilized at concentration equals 15 nM. Transfection was performed with Lipofectamine™ 3000 Transfection Reagent (Thermo Fisher Scientific, L3000001) according to the manufacturer's protocol.

To assess the effect of miRNAs inhibition on IL-11-induced fibrosis, rCFs were first transfected with miRNA-27b-5p (Cat. Number MH20018) or miRNA-497-5p (Cat. Number MH11293) mirVana™ inhibitors, or with universal scramble control nucleotides (Horizon Discovery, D-001810-10-20) at a concentration of 5 nM using Lipofectamine™ 3000 Transfection Reagent (Thermo Fisher Scientific, L3000001). Twenty-four hours after transfection, cells were treated with IL-11 (5 ng/mL) or an equal volume of PBS and incubated for an additional 24 hours before harvest for downstream analyses.

To assess the impact of **EGLN1 overexpression** on **IL-11-induced fibrosis**, rCFs were transfected with a custom **EGLN1-overexpressing plasmid** (VectorBuilder) at a concentration of 500 nM using **Lipofectamine™ 3000 Transfection Reagent** (Thermo Fisher Scientific, L3000001). After 24 h, cells were treated with **IL-11** (5 ng/mL) or an equal volume of PBS and incubated for an additional 24 h. Cells were then harvested for downstream analyses.

## Immunostaining of cardiac fibroblasts

CFs were fixed with 4% paraformaldehyde, followed by permeabilization with 0.05% Triton X-100 diluted in PBS. Non-specific binding was prevented by blocking fixed cells with 5% (w/v) BSA for 1 hour at room temperature. After that, cells were stained overnight at 4 °C with primary antibodies diluted in 5% BSA in PBS: PDGFRα rabbit monoclonal antibody (ab203491) (1:1000 v/v), α-SMA mouse monoclonal DAKO antibody (M0851) (1:250 v/v), and vimentin chicken polyclonal antibody from Invitrogen (PA1-16759) (1:500 v/v). To control for background signal and cells autofluorescence, negative control samples were processed in parallel under identical conditions with omission of the primary antibodies. The next day, cells were washed three times with PBS and incubated for 1 hour with the following secondary antibodies: anti-rabbit goat AlexaFluor-488 from Invitrogen (A11008) or anti-mouse donkey polyclonal AlexaFluor-488 from Invitrogen (A21202), and anti-chicken goat polyclonal AlexaFluor-546 from Invitrogen (A11040) (diluted in PBS 1:250 v/v). After that, cells were washed in PBS three times and mounted onto the labeled slides, using hard-set mounting medium (ProlongTM Gold Antifade reagent with DAPI). Sections were stored at 40 °C. In selected experiments, post-MI HF cardiac fibroblasts, characterized by a high proportion of α-SMA-positive cells, were used as positive controls to confirm antibody performance and staining specificity.

**Image acquisition and analysis for immunostaining in cell culture**

Images were collected with the optical system [Nikon Eclipse Ti with pE-4000 light source (Cool LED) and ORCA-Flash 4 camera (Hamamatsu)]. By using 20x magnification ~200 cells were imaged per condition in total with ~10 cells per image. Image processing was done in Fiji (ImageJ). To distinguish α-SMA- or PDGFRα-positive cells from negative ones, images were thresholded to a control condition. Raw images in DAPI and vimentin channels were uploaded in ImageJ and converted to 16-bit images, where cells were manually highlighted and mean fluorescence for each cell was detected in the α-SMA (red) channel with the help of the ROI Manager. After that, mean fluorescence from each cell was used for distribution analysis, where the 90th percentile was chosen as a threshold value for all conditions. Fiber morphology was also considered an important criterion for α-SMA-positive cell detection.

## RNA isolation and reverse transcription polymerase chain reaction

Total RNA isolation from cell cultures and LV biopsies was performed using TRIzol™ Reagent (Thermo Fisher Scientific, 15596026) following the manufacturer’s protocol

miRNAs and small RNAs were isolated from plasma and plasma EVs of AS patients and healthy donors by using Nucleospin miRNA Plasma kit (Macherey Nagel), after the addition of cel-miR-39 spike-in for normalization (5 pmol/µL) (Thermo Fisher Scientific).

The GoScript™ Reverse Transcriptase kit (Promega, A5001) was used for mRNA cDNA synthesis according to the manufacturer’s protocol. RNA samples were supplemented with random primers and oligo(dT) primers and incubated at 70 °C for 5 minutes. Reverse transcription was performed in an Eppendorf Mastercycler PRO S 6325 Thermal Cycler under the following conditions: 25 °C for 5 minutes, 42 °C for 60 minutes, and enzyme inactivation at 70 °C for 15 minutes, followed by a final hold at 4 °C. RT-qPCR was performed using the GoTaq® qPCR kit (Promega, A6001) on an Applied Biosystems® QuantStudio™ 6 Flex Real-Time PCR System with an initial denaturation at 95 °C for 3 minutes, followed by 40 cycles of 95 °C for 10 seconds and 60 °C for 30 seconds. Genes analyzed are listed in Table S4.

## MicroRNAs and U6 small nuclear RNA were polyadenylated and reverse transcribed using the TaqMan™ MicroRNA Reverse Transcription Kit (Thermo Fisher Scientific, 4366596). Reverse transcription was performed in an Eppendorf Mastercycler PRO S 6325 Thermal Cycler under the following conditions: 16 °C for 30 minutes, 42 °C for 30 minutes, and 85 °C for 5 minutes, followed by a final hold at 4 °C. RT-qPCR for miRNAs was performed using Applied Biosystems™ TaqMan™ Fast Universal PCR Master Mix (Thermo Fisher Scientific, 4352042) on an Applied Biosystems® QuantStudio™ 6 Flex Real-Time PCR System. Cycling conditions consisted of an initial denaturation at 95 °C for 20 seconds, followed by 40 cycles of 95 °C for 1 second and 60 °C for 20 seconds. MicroRNAs and U6 small nuclear RNA analyzed are listed in Table S5.

**Bioinformatic miRNA target prediction analysis to identify miRNAs regulated by IL-11 in cardiac fibroblasts**

Figure 2A offers a graphical representation of the bioinformatic miRNA target prediction pipeline adopted to predict miRNAs regulating cardiac fibrosis. IL-11 induces fibrosis and aging acting via a non-canonical pathway initiated by ERK^6,56,57^ that is linked to canonical TGF-β signaling.^6,56,57^ Pro-fibrotic effect was shown to be pre-dominantly through Erk-cascade.^6,56,57^ For this reason, we further address IL-11 signaling pathway as IL-11 (ERK). To identify the miRNAs expressed in rat and human CFs that could regulate these linked signaling pathways, we designed a multi-step pipeline. First, we analyzed a publicly available dataset (GEO dataset GSE76175) and identified 728 miRNAs expressed in rat CFs and extracellular vesicles derived from these cells. From the KEGG pathway database,^58^ we identified a total of 410 mRNAs involved in the IL-11 (ERK) and TGFβ1 signaling pathways. By using mirTarbase we checked how many of these miRNAs have experimentally supported targets by RT-qPCR or microarray within two pathways (226 miRNAs).^59^ In other words, we selected 226 miRNAs that are more likely to affect signaling cascades. The remaining 502 miRNAs holding higher novelty potential were ranked according to expression level in CFs, and the top 25% most abundant miRNAs (127 miRNAs) were selected for further analysis alongside the 226 validated candidates, resulting in a total of 353 candidate miRNAs. Next, we examined potential binding of these 353 miRNAs to genes within the IL-11 (ERK) and TGFβ1 pathways using three prediction tools: TargetScan, miRWalk, and miRcode.^60–62^ Only miRNAs predicted to target genes in both pathways across all three databases were considered (68 miRNAs). Additional selection criteria required that the binding sites are conserved between rat and human.^63^ These stringent steps allowed us to minimize false positives and increase confidence in the predictions. Finally, we obtained a set of 7 miRNAs with predicted affinity to 3 or more targets within the IL-11 (ERK) and TGFβ1 signaling pathways (≥3 targets). This refined set of miRNAs was carried forward for functional analysis.

## RNA-sequencing

RNA-seq was conducted as reported.^41^ Briefly, total RNA was isolated with TRIzol from human dilated cardiomyopathy CFs (N = 3) treated with IL-11 (5 ng/mL for 24 hours) and from non-stimulated fibroblasts (N = 3). Isolation purity was evaluated by spectrophotometry, with RNA samples with a 260/280 of > 2 and a 260/230 of > 2 taken forward. Ribosomal RNA was depleted using the RiboMinus™ Eukaryote Kit (Thermo Fisher Scientific). The Imperial BRC Genomics facility performed RNA-seq. Differential expression analysis was performed using EdgeR^64^ with default settings. The Benjamini-Hochberg method was used for false discovery rate (FDR) adjustment, and genes with an adjusted p-value < 0.05 were considered significantly differentially expressed.

## Shortlisting of genes in the IL-11 signaling pathway regulated by miRNA-27b-5p and miRNA-497-5p

Targets of miRNA-27b-5p (number of targets = 596) and miRNA-497-5p (number of targets = 140) were filtered by identifying their expression in CFs after IL-11 stimulation. Next, over-representation analysis for the remaining candidate targets was performed by WebGestalt^65^ with default settings. The most enriched pathway was determined by p-values (p-value < 0.05). Targets from this pathway were used to plot the gene network in Cytoscape.^66^

**HEK293FT Cell Culture**

HEK293FT cells (Invitrogen^TM^) were used for the dual luciferase reporter assays. Cells were maintained in high-glucose DMEM supplemented with 10% FBS and 1% penicillin-streptomycin. Cells were incubated at 37 °C with 5% CO_2_. Cells were grown for three days until they reached 100% confluence, prior to luciferase assay experiments.

## 3’UTR luciferase assays

We adapted a previously described protocol to demonstrate the direct binding and expressional control exerted by miRNA-27b-5p and miRNA-497-5p on EGLN1 emerging from our bioinformatic analyses.^67^ HEK293FT cells were seeded in 96-well plates and transfected with either wild-type (WT), mutant (MUT) target constructs, or vector alone (V.A.) in combination with miRNA control (Scramble - GeneCopoeia^TM^ CmiR0001-MR04), *Homo sapiens* miR-27b stem-loop (GeneCopoeia^TM^ HmiR0145-MR04) or with *Homo sapiens* miR497 stem loop (GeneCopoeia^TM^ HmiR-0271-MR04) and with pRL-null renilla luciferase. Luminescence was measured using a 1420 Victor³ Multilabel Counter (PerkinElmer).

## Clinical samples

The clinical aspects of our study included four cohorts (summarized in Tables S1 and S2): 1) AS discovery cohort (blood plasma and tissue); 2) healthy volunteers (blood plasma); 3) donor heart (tissue); 4) AS validation cohort (blood plasma). The discovery AS cohort was prospectively recruited (2018 to 2021) at the Policlinico G.B. Rossi, Verona, Italy, and the validation AS cohort was recruited in 2024 at the Hammersmith Hospital, London, UK. AS patients in both the discovery and validation cohorts had symptomatic severe, high-gradient AS confirmed by echocardiogram, mean gradient ≥ 40 mmHg, peak velocity ≥ 4.0 m/s, valve area ≤ 1.0 cm^2^ (or ≤ 0.6 cm^2^/m^2^), trileaflet aortic valve and preserved left ventricular ejection fraction (LVEF) ≥ 55%. Exclusion criteria were: (1) bicuspid anatomy, (2) low flow-low gradient AS, (3) history of coronary artery disease or significant coronary artery disease with an indication to surgical revascularization concomitant to SAVR, (4) impaired left ventricular function, (5) history of arrhythmia, including atrial fibrillation, (6) chronic kidney disease, and liver dysfunction. Patients with a significantly hypertrophic septum underwent concomitant septal myectomy. Blood plasma and LV samples (frozen or formalin-fixed) were collected. Detailed characteristics and comorbidities of the AS patients are presented in Table S3. Control blood donors were recruited at IRCCS Policlinico San Donato, Milan, Italy. Donor hearts were provided by the Department of Heart Failure and Transplantology, Cardinal Stefan Wyszyński Institute of Cardiology, Warsaw, Poland.

## 2DE speckle-tracking strain analysis of AS patient echocardiogram

Standard 2D images of the LV of AS validation cohort patients were obtained in the apical 2-, 3- and 4-chamber views. Dedicated views of the left atrium (LA) were acquired with care taken not to foreshorten the LA chamber. Images were recorded at frame rates between 50-90 frames per second (fps) to ensure optimal software analysis. Offline speckle-tracking analysis of global longitudinal strain (GLS) and LA strain was performed using AutoStrain LV/LA software (TomTec Imaging Systems). Endocardial border tracking was performed automatically. Tracking quality was visually verified, and segments that failed initial tracking were manually adjusted. Segments that could not be properly or were inadequately tracked after manual adjustment were rejected. If ≥3 LV segments were inadequate, the patient’s strain data were excluded from the final analysis. The following 2D endocardial speckle-tracking echocardiography (STE) values were obtained: peak-systolic LV GLS, and end-systolic LA reservoir, conduit, and contractile strain.

**Histology and morphometric analysis of the human samples**

Human heart samples (AS discovery cohort and donor heart tissue) were fixed and embedded in paraffin according to standard protocols. Paraffin-embedded tissue sections were cut at a thickness of 10 µm and ECM was stained in tissue histology sections of AS (N = 11) and control samples (N = 10) using a Picro Sirius Red Stain Kit. For immunofluorescence staining, sections were blocked in 5% BSA supplemented with 10% donkey and goat serum at room temperature for 1 hour. After that, the following primary antibodies were used: anti-collagen I (ab34710, Abcam Cambridge, UK) (1:200 v/v dilution in 5% BSA in PBS with 0.05% Tween) and anti-alpha-sarcomeric actin clone 5C5 (#2172, Sigma-Aldrich, Merck Darmstadt, Germany) (1:500 v/v dilution in 5% BSA in PBS with 0.05% Tween). Negative control sections were processed in parallel under identical conditions with omission of the primary antibodies to assess nonspecific signal and tissue autofluorescence. The next day sections were incubated at room temperature for 1 hour with secondary antibodies: donkey anti-rabbit Alexa Fluor 555 (A-31572) and Alexa Fluor 488 goat anti-mouse (ab150121), both diluted 1:250 v/v in PBS with 0.05% Tween.

**Histology: image acquisition and collagen quantification**

Sections were stained with Picro Sirius Red Stain Kit to quantify ECM deposition, according to the manufacturer’s protocol. Images were collected by a Zeiss AxioLab-A1 microscope (Zeiss, Oberkochen, Germany) equipped with a True Chrome HD II S camera (Tiesse Lab, Cassano d’Adda (MI), Italy) in the brightfield with a 2.5x objective at a resolution equal to 3264x1836 pixels. The quantification of the areas of ECM deposition across entire sections was performed with Fiji (ImageJ) software.

For the quantification of Collagen I area in LV biopsies from patients, images were obtained using the high-resolution DeltaVision Ultra microscope (Cytiva, Danaher Corporation Life Sciences company) with a 20X/0.75 objective, automatic stitching, and a z-stack step of 2.5 µm. Images were acquired and analyzed by DeltaVision Ultra's advanced software.

## Plasma extracellular vesicle analysis

Citrate plasma was prepared from peripheral blood of the AS discovery cohort (N = 23), healthy donor cohort, used during the discovery phase (N = 10) and AS validation cohort (N=14), and stored at -80^0^C. Plasma EVs were isolated by size exclusion chromatography, using the Exo-spin Blood Kit following the manufacturer’s protocol (CELL Guidance Systems). The quality of the EVs preparation was validated using transmission electron microscopy with 2% uranyl acetate staining (transmission electron microscope TALOS L120C Thermo Fisher Scientific at 120 kV).

**Blood plasma treatment with proteinase K**

The protocol was adapted from the previous study of *Arroyo et al.*^34^ Recombinant, molecular-grade, DNase/RNase–free proteinase K (Worthington Biochemical Corporation, USA) was prepared in RNase-free water (50 mg/mL). Three samples of plasma from a donor were prepared on ice. At 0 min, 0 mg/mL or 5 mg/mL proteinase K was added to the plasma and the samples were incubated at 55 °C for 30 minutes. The control sample was left on ice for 30 minutes (0 mg/mL proteinase K added). Aliquots were removed at time indicated and denatured in MLP lysis buffer from Nucleospin miRNA Plasma kit (Macherey-Nagel), followed by immediate RNA isolation, according to manufacturer’s protocol**.** Cel-miR-39-5p spike-in oligonucleotides (5 pmol/µL) (Thermo Fisher Scientific) were added to the denatured samples.

## Statistical analysis

Statistical analysis was performed with Origin 8 Pro and RStudio. The specific tests used are indicated in the figure legends. For comparisons between two groups Mann-Whitney U test was applied. Comparisons among multiple groups were performed using one-way ANOVA followed by Tukey’s multiple comparisons test. Correlations were assessed using Pearson’s correlation coefficient following confirmation of normal distribution. N - number of patients/animals, n - number of technical repeats. Data are presented as Mean ± SEM. P-values < 0.05 were considered statistically significant.

**Supplementary Figures’ legends**

**Figure S1.** **Myocardial infarction induced by coronary artery ligation decreases the fractional shortening and ejection fraction in rats.** **A)** Representative echocardiography pictures of M-mode images of age-matched sham-operated rats **(i)** and rats with MI induced by coronary artery ligation **(ii)**.  **B)** Fractional Shortening (%) and **C)** Ejection Fraction (%) of age-matched sham operated (white) and MI rats (grey), N = 4. Mann-Whitney U test was performed in order to estimate statistical significance.

**Figure S2. Confirmation of fibroblasts culture purity by PDGFRα and vimentin staining. A)** Representative image of rat CFs stained against vimentin (red), PDGFRα (green), and DAPI (blue) was used to detect nuclei. Four fields of view were imaged per coverslip using a 20x magnification with ~ 12-20 cells per image. **B)** Percentage of PDGFRα and vimentin positive cells across all images is presented as a pie chart: dark grey ─ PDGFRα-positive cells, light grey ─ PDGFRα-negative cells.

**Figure S3. Rat CFs isolation: IL-11 and post-MI HF *in vitro* models of fibrosis. A)** CFs were isolated following enzymatic digestion of the LV of rats at 16 weeks post-MI and sham-operated control rat. MI was induced in anaesthetized male Sprague-Dawley 3-month-old rats by permanent LAD ligation for 16 weeks. **B)** CFs were isolated following enzymatic digestion of the LV of healthy rats. **A)-B)** Cells were cultured in DMEM +10% Fetal bovine serum +1% antibiotics at 37 °C with 5% CO_2_. Following this, cells were seeded equally (~100 000 cells/well) in 12-well plates for further experiments at the passage 3-5. **B)** 24 hours after rCFs were seeded, they were treated with IL-11 (5 ng/mL) or the equal volume of PBS for 24 hours.

**Figure S4. Mmp2 expression.** RT-qPCR analysis Mmp2 mRNA expression (normalized to GAPDH: A or UBC: B, C) (N = 6; n = 3). Mmp2 expression in rCFs **A)** stimulated with IL-11 (5 ng/mL, 24 hours) **(i)** or from post-MI HF rCFs **(ii).** Transfection of healthy rCFs **(i)** or post-MI HF rCFs **(ii)** with scramble nucleotides (white bar), miRNA-27b-5p or -497 mimic (light grey) and inhibitor (dark grey) at 5nM concentration. Experiments realized for the two gain- and loss-of-function protocols: **B)** miRNA-27b-5p and **C)** miRNA-497-5p (N = 6, n = 3). Mann-Whitney U test was used to determine significance between control and group of interest.

**Figure S5. Inhibition of either miRNA-27b-5p or miRNA-497-5p suppresses IL-11 induced fibrosis in rCFs.** Healthy rCFs were transfected with scramble nucleotides (white, light grey bars), miRNA-27b-5p inhibitor (grey bars), or miRNA-497-5p inhibitor (dark grey bars) at 5 nM, and 24 h later treated with IL11 (light grey, grey, dark grey bars) or PBS (white bars) at 5 ng/ml for additional 24 h. **A)** Representative images i)-iv) stained with vimentin (red) and α-SMA (green) and DAPI (blue). **B)** Quantification of α-SMA positive cells (%) (N = 7, n = 2). ~300 cells were quantified for each condition, with 10-20 cell/image. **C)-D)** RT-qPCR analysis of miRNA/mRNA of interest: expression was normalized to U6 RNA expression and further referred to the scramble + PBS condition, while mRNA expression was normalized to UBC RNA expression. Expression of **C)** miRNA-27b-5p (i) and miRNA-497-5p (ii), **D)** α-SMA (i), Col1a1 (ii), Postn (iii) and Adamts5 (iv) (N = 8, n = 3). One-way ANOVA followed by Tukey’s multiple comparisons test was used to assess significance between the groups.

**Figure S6. miRNA-27b-5p regulates IL-11-induced cardiac fibrosis through targeting EGLN1. A)** Healthy rCFs were transfected with scramble nucleotides (white, light grey bars) or miRNA-27b-5p inhibitor (grey bars) at 5 nM, and 24 h later treated with IL11 (light grey, grey bars) or PBS (white bars) at 5 ng/ml for additional 24 h. Expression of Egln1 was assessed with RT-qPCR analysis and normalized to UBC RNA expression (N = 8, n = 3). **B)-C)** Healthy rCFs were transfected with Egln1 overexpressing (Egln1 OE) plasmid (grey and dark grey bars) at 500 nM, and 24 h later treated with IL11 (light grey, grey, dark grey bars) or PBS (dark grey bars) at 5 ng/ml for 24 h. **B)-C)** RT-qPCR analysis of mRNA of interest: expression was normalized to Ubc mRNA expression and further referred to the scramble-treated control condition. Expression of **B)** Egln1, **C)** α-SMA (i), Postn (ii) (N = 8, n = 3). One-way ANOVA followed by Tukey’s multiple comparisons test was used to assess significance between the multiple groups, Mann-Whitney U test was used to assess significance between 2 groups.

**Figure S7. miRNA-27b-5p overexpression and inhibition in human CFs.** **A)-F)** Transfection of human cardiac fibroblasts (isolated from 1 patient, done in 4 replicates) with scramble nucleotides (white), miRNA-27b-5p mimic (light grey) and inhibitor (dark grey) at concentration 15 nM. **A)** Representative images of human CFs transfected with nucleotides and stained after 24 hours against vimentin (red) and α-SMA (green), DAPI (blue) was used to detect nuclei. Six fields of view were imaged per coverslip using a 20x magnification with ~ 20 cells for image. **B)** Bar chart of the percentage of α-SMA positive cells in human donor. Expression in human donor CFs for following genes **C)** miRNA-27b-5p, **D) (i)** α-SMA, **(ii)** EGLN1**,** and **(iii)** HIF2α is present as a bar chart. One-way ANOVA was applied, followed by Tukey’s Mean Comparison to estimate statistical significance.

**Figure S8. miRNA-27b-5p and miRNA-497-5p are upregulated *in vivo* in the heart of Tg-Il11 mice. A)** Breeding scheme used to produce Tg-Il111 mice and their littermate controls. **B)** Schematic representation of the targeted overexpression of Il11 in cardiomyocytes. In the Rosa26-Il11 transgene, a floxed cassette containing neomycin (neo) resistance and stop elements is located before the murine Il11 transgene cassette. This cassette undergoes tamoxifen-triggered, Cre-mediated recombination when crossed with the α-MHCMerCreMer (MCM) mouse. **C)-D), F)-H)** RT-qPCR analysis of mRNAs and miRNAs expression in the LV tissues of Tg-Il11 mice (grey, N = 6) and littermate controls (white, N = 4). The expression of miRNA is normalized to U6 RNA, the expression of mRNAs is normalized to Ubc RNA. **C)** IL-11 expression in LV tissues of Tg-Il11 mice, **D) (i)** miRNA-27b-5p and **(ii)** miRNA-497-5p expression in LV tissues of Tg-Il11 mice. **E)** Pearson correlation between IL11 and **(i)** miRNA-27b-5p or **(ii)** miRNA-497-5p expression in LV tissues of Tg-Il11 and control mice. **F)** Tgfβ1 expression in LV tissues of Tg-Il11 mice. **G) (i)** Egln1, **(ii)** Egln2, **(iii)** Hif1α expression in LV tissues of Tg-Il11 mice. **H) (i)** Col1a1, **(ii)** αSMA, **(iii)** Mmp2 expression in LV tissues of Tg-Il11 mice. Mann-Whitney U test was performed in order to estimate statistical significance.

**Figure S9. Cardiac parameters of TAC and sham mice post-surgery. A-i)** Assessment of the peak gradient. The peak gradient was measured 7 days after surgery by ultrasound imaging in Power Doppler Mode. **B)-G)** Two-dimensional short-axis images and M-mode tracings were recorded at the level of papillary muscles. The echocardiographic parameters were obtained from TAC mice (N = 4, grey) and sham-operated mice (N = 6, white) at 60 days post-surgery, with sham-operated mice serving as reference values. **B-i**) The percentage of fractional shortening; **B-ii)** left ventricle ejection fraction (LVEF); **C)** LV mass; **D-i)** LV volume in diastole; **D-ii)** LV volume in systole; **E-i)** LV anterior wall (LVAW) in diastole; **E-ii)** LVAW in systole; **F-i)** LV internal diameter (LVID) in diastole; **F-ii)** LVID in systole; **G-i)** LV posterior wall (LVPW) in diastole; **G-ii)** LVPW in diastole. Mann-Whitney U test was performed to estimate statistical significance.

**Figure S10. miRNA-27b-5p and miRNA-497-5p are upregulated in the LV of TAC mice.** RT-qPCR analysis of mRNAs and miRNAs expression in the LV tissues of TAC mice (grey, N = 4) and sham-operated controls (white, N = 6). The expression of miRNA is normalized to U6, the expression of mRNAs is normalized to Ubc. **A)** IL-11 expression in LV tissues of TAC mice, **B) (i)** miRNA-27b-5p and **(ii)** miRNA-497-5p expression in LV tissues of TAC mice. **C)** Pearson correlation between IL11 and **(i)** miRNA-27b-5p or **(ii)** miRNA-497-5p expression in LV tissues of TAC and control mice. **D)** Tgfβ1 expression in LV tissues of TAC mice**. E) (i)** Egln1 **(ii)** Egln2, **F)** Ctgf expression in LV tissues of TAC mice. **G) (i)** Col1a1, **(ii)** α-SMA, **(iii)** Mmp2 expression in LV tissues of TAC mice. Mann-Whitney U test was performed to estimate statistical significance.

**Figure S11. miRNA-27b-5p, miRNA-497-5p, EGLN1 and EGLN2 expression in LV of AS patients with Hypertension, Dyslipidemia and in old patients. A)-L)** RNA was isolated with TRIzol from (N = 15) LV from AS patients and (N = 14) controls that died from non-CVD reasons. **A)-B), E)-F), I)-J)** RT-qPCR on miRNAs. **C)-D), G)-H), KL)** RT-qPCR on EGLN1/2. **A)-D)** miRNA-27b-5p, miRNA-497-5p-5p, EGLN1/2 expression in LV of AS patients with hypertension (N = 11) compared with AS patients without hypertension (N = 2). **E)-H)** miRNA-27b-5p, miRNA-497-5p-5p, EGLN1/2 expression in LV of AS patients with dyslipidemia (N = 7) compared with AS patients without dyslipidemia (N = 6). **I)-L)** Pearson correlation of miRNA-27b-5p, miRNA-497-5p-5p, EGLN1/2 expression in LV of AS with age (N = 15). Mann-Whitney U test was performed to estimate statistical significance.

**Figure S12. miRNA-27b-5p and miRNA-497-5p expression in plasma and EVs of AS patients with Hypertension, Dyslipidemia and in old patients. A)-L)** miRNA from (N = 23) EVs from plasma of AS patients and compared with miRNA isolated from (N = 10) healthy donors. miRNA of interest expression was normalized to cel-miR-39 and further referred to a control group. **A)-D)** miRNA-27b-5p in plasma, miRNA-497-5p-5p in plasma, EVs-miRNA-27b-5p in EVs from plasma and EVs-miRNA-497-5p-5p in EVs from plasma expression in AS patients with Hypertension (N = 20) and compared with AS patients without Hypertension (N = 4). **E)-H)** miRNA-27b-5p in plasma, miRNA-497-5p-5p in plasma, EVsmiRNA-27b-5p in EVs from plasma and EVs-miRNA-497-5p-5p in EVs from plasma expression in AS patients with Dyslipidemia (N = 12) and compared with AS patients without Dyslipidemia (N = 12). **I)-L)** Pearson correlation between AS patients age and miRNA-27b-5p in plasma, miRNA-497-5p-5p in plasma, EVs-miRNA-27b-5p in EVs from plasma and EVs-miRNA-497-5p-5p in EVs from plasma expression in AS patients (N = 22). Mann-Whitney U test was performed in order to estimate statistical significance.

**Figure S13. Proteinase K digestion at 55 °C selectively sensitizes plasma miRNAs to degradation by endogenous RNases. A)** PCR analysis of **(i)** let-7a-5p and **(ii)** miRNA-16-5p expression normalized to cel-miR-39-5p expression in blood plasma. **B)** PCR analysis of **(i)** miRNA-27b-5p and **(ii)** miRNA-497-5p expression normalized to cel-miR-39-5p expression in blood plasma. Mann-Whitney U test was performed to estimate statistical significance.

**Supplemental Figures**


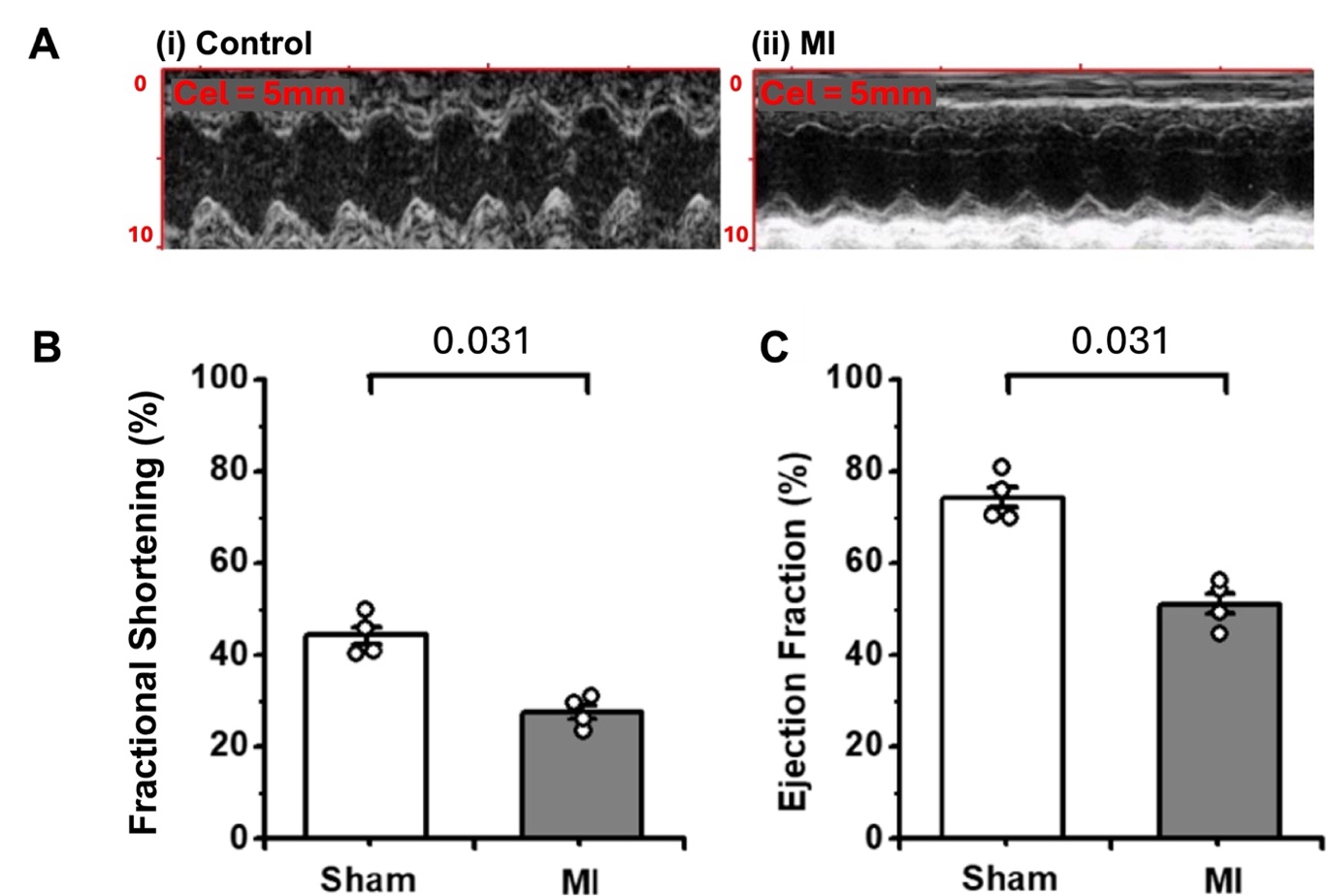


**Figure S1. Myocardial infarction induced by coronary artery ligation decreases the fractional shortening and ejection fraction in rats.**

**
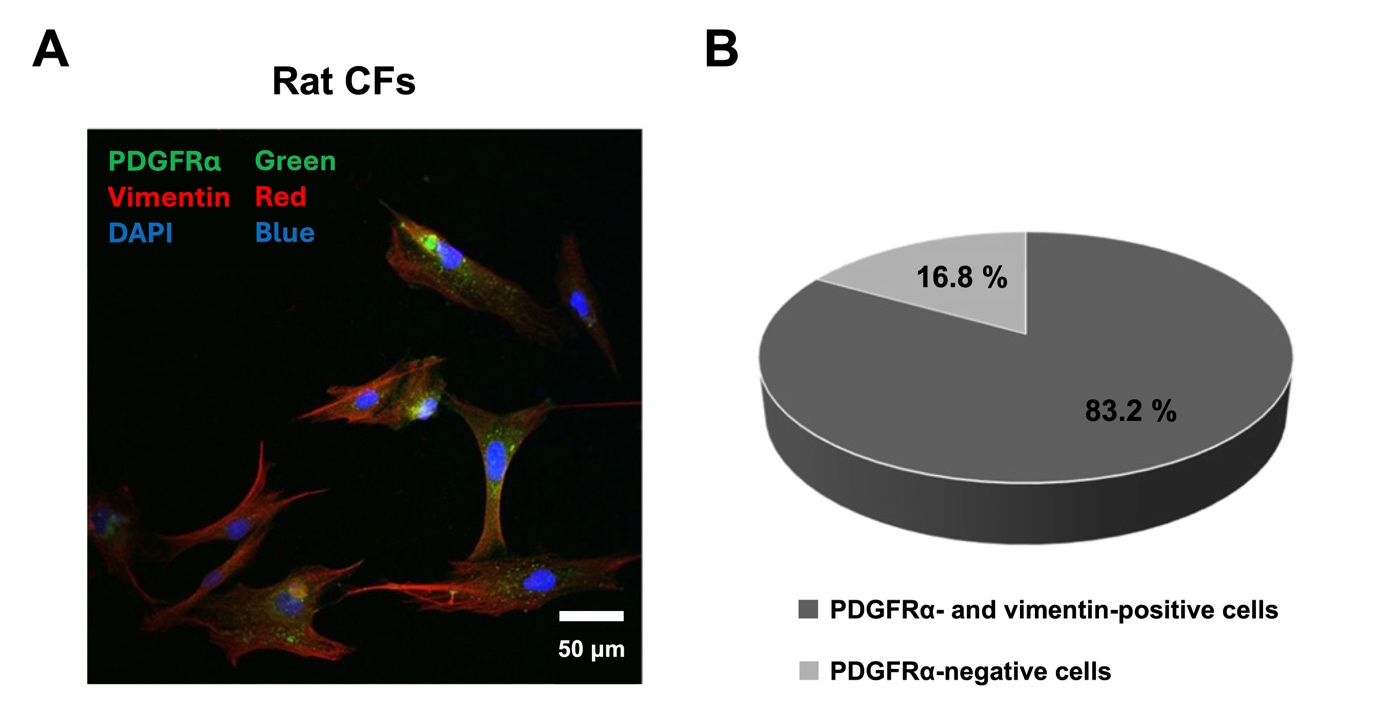
Figure S2. Confirmation of fibroblasts culture purity by PDGFRα and vimentin staining.**

**
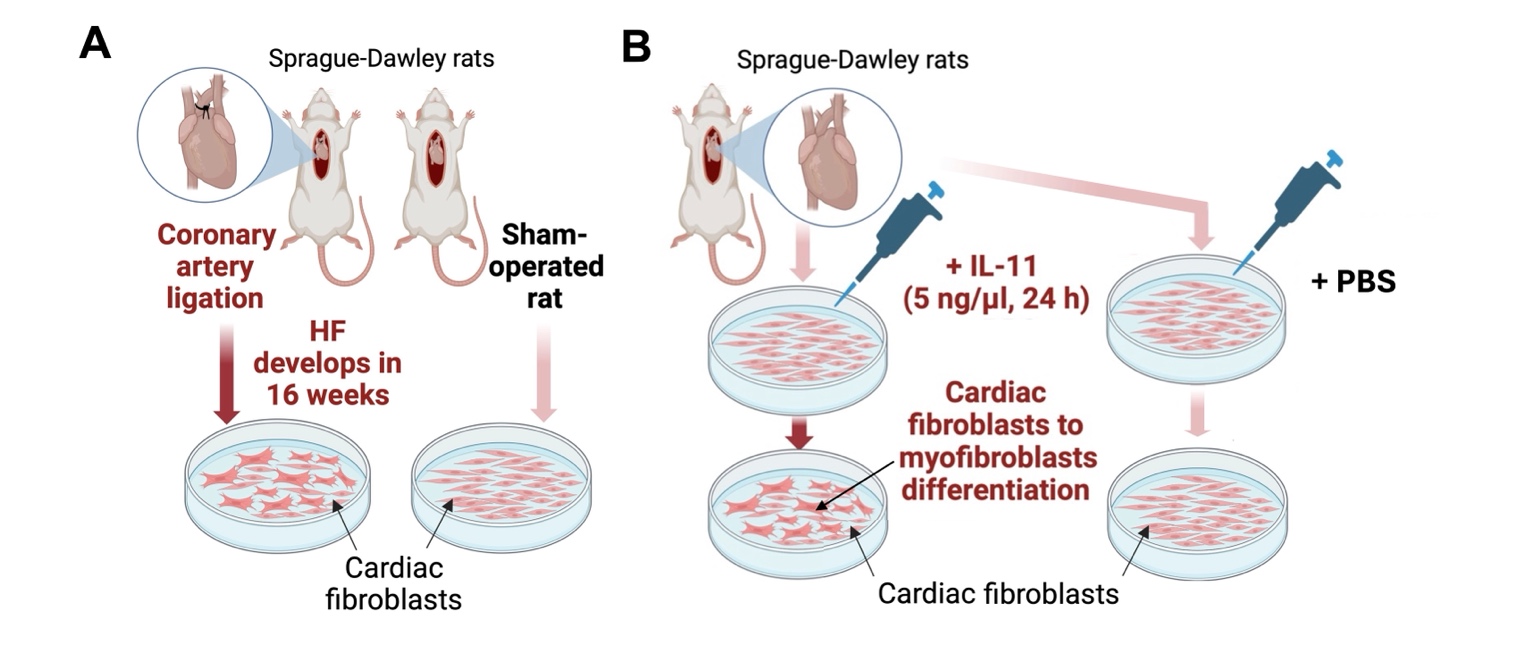
Figure S3. Rat CFs isolation: IL-11 and post-MI HF in vitro models of fibrosis.**

**
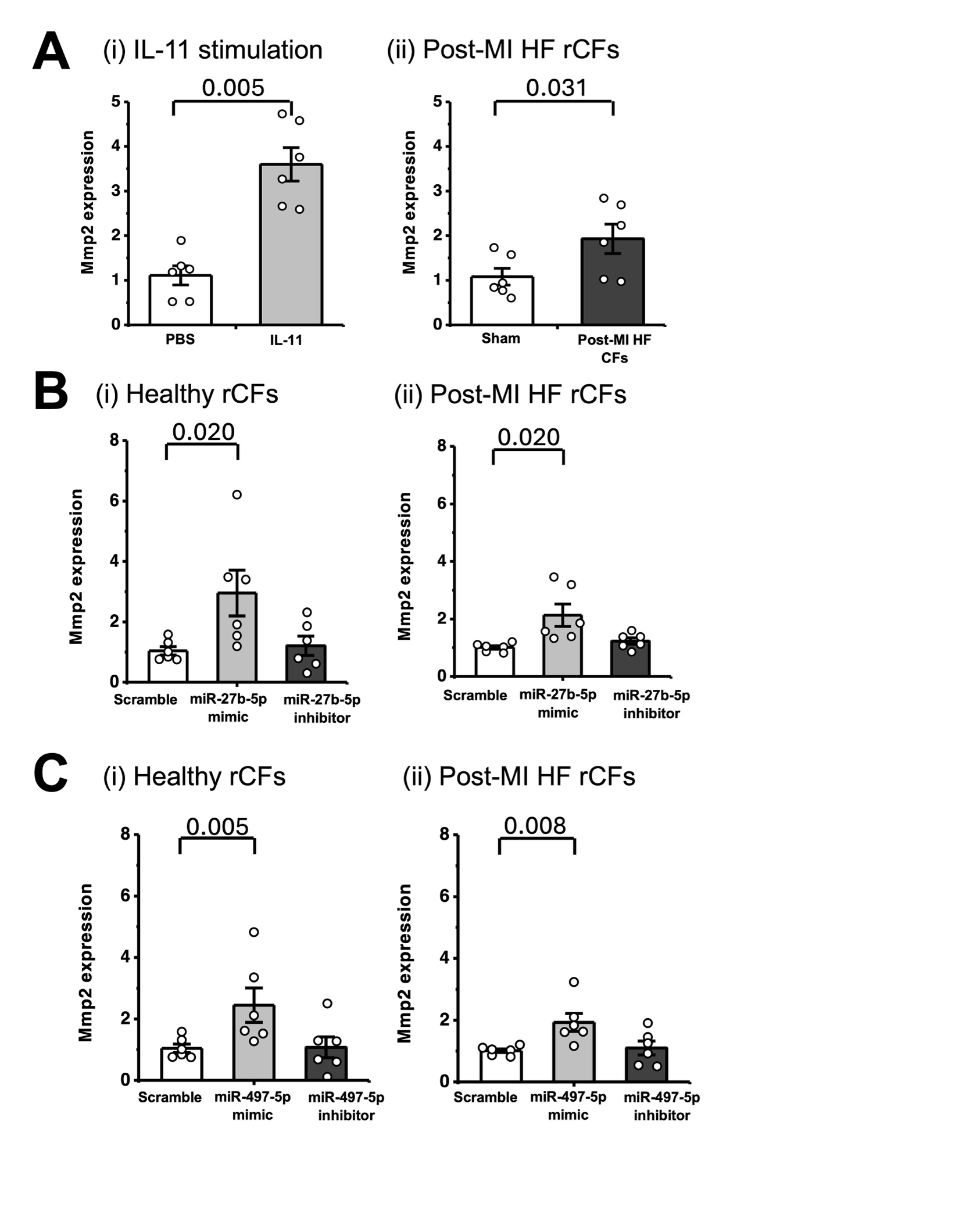
**

**Figure S4. Mmp2 expression.**

**
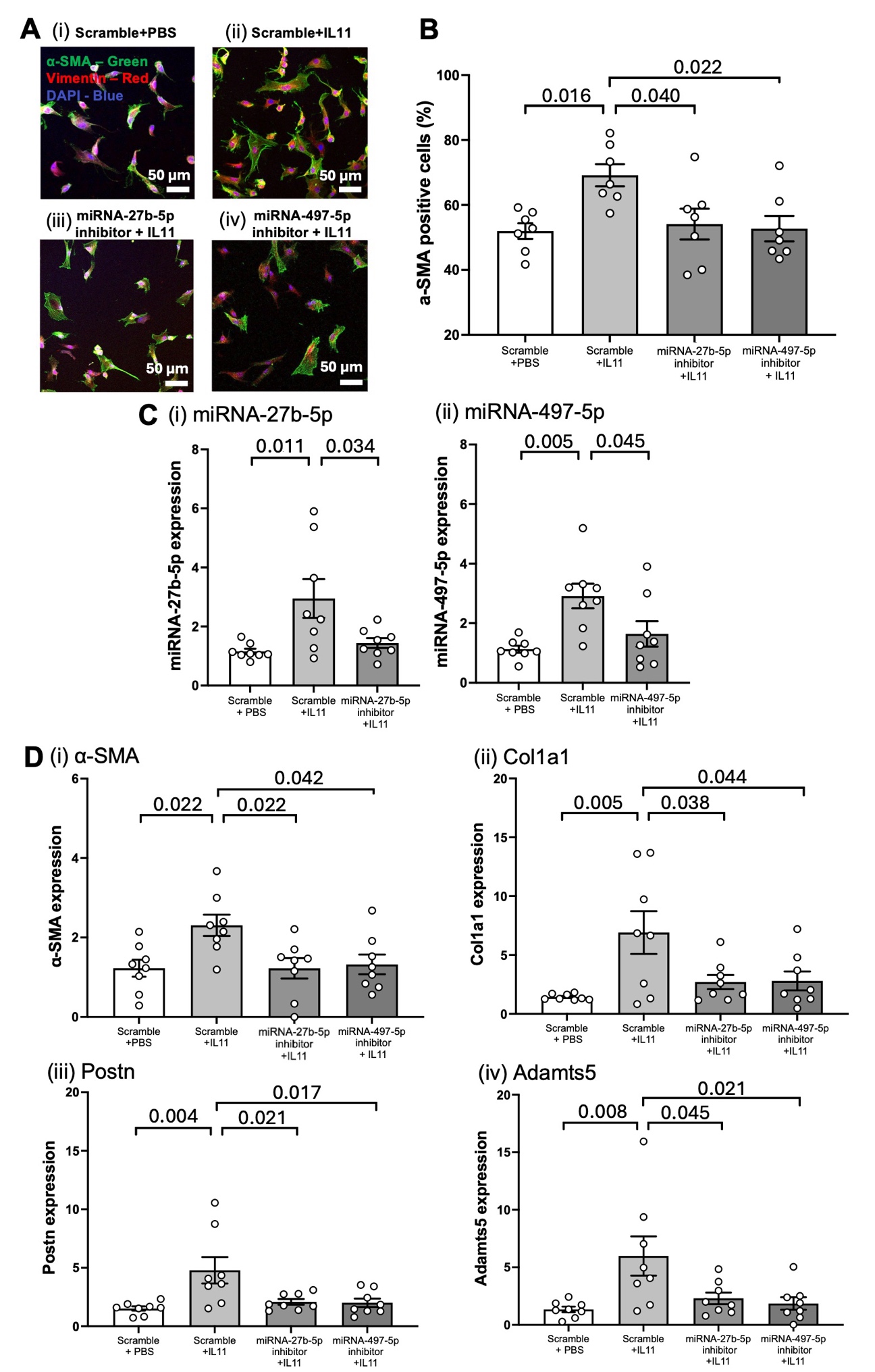
**

**Figure S5. Inhibition of either miRNA-27b-5p or miRNA-497-5p suppresses IL-11-induced fibrosis in rCFs.**

**
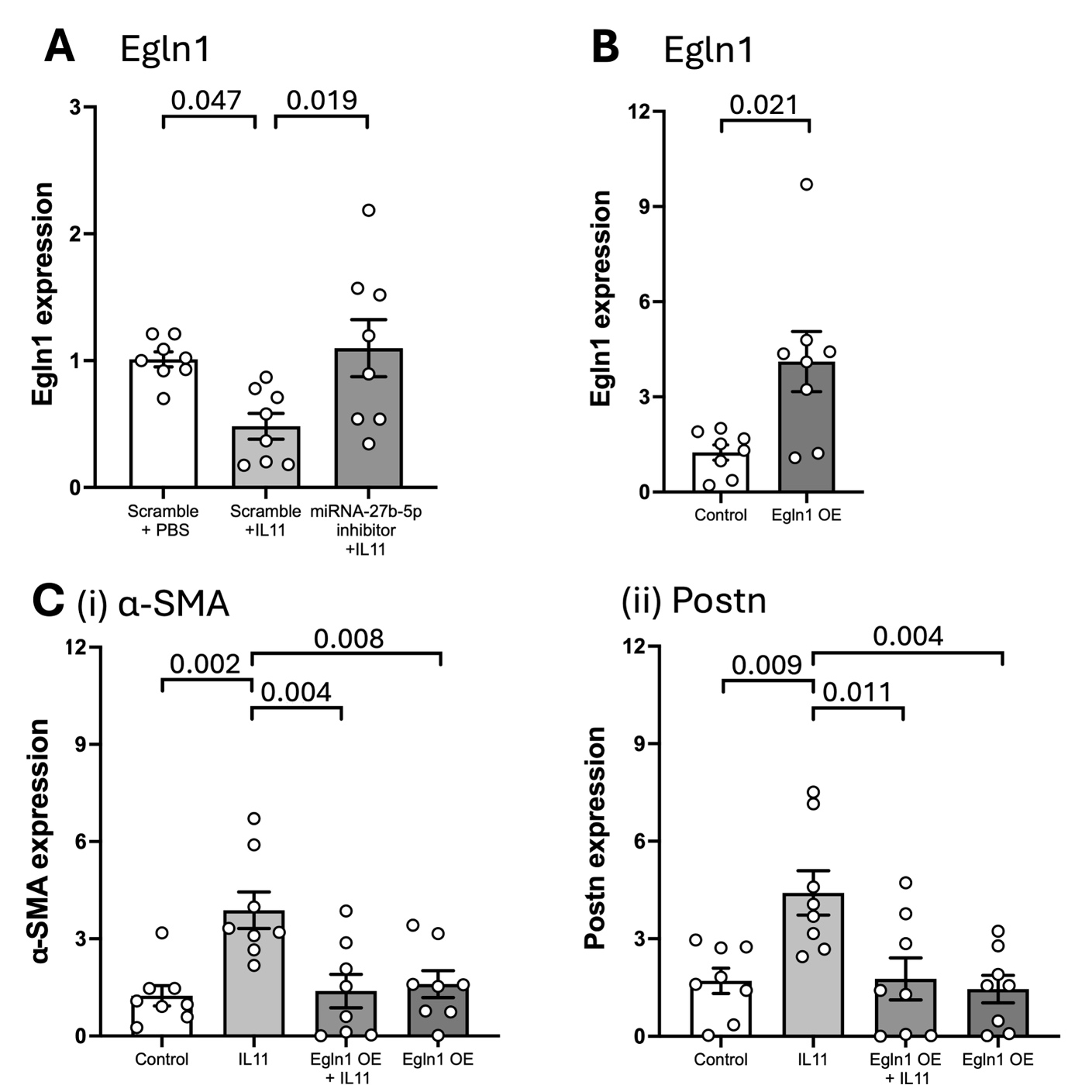
**

**Figure S6. miRNA-27b-5p regulates IL-11-induced cardiac fibrosis through targeting EGLN1.**

**
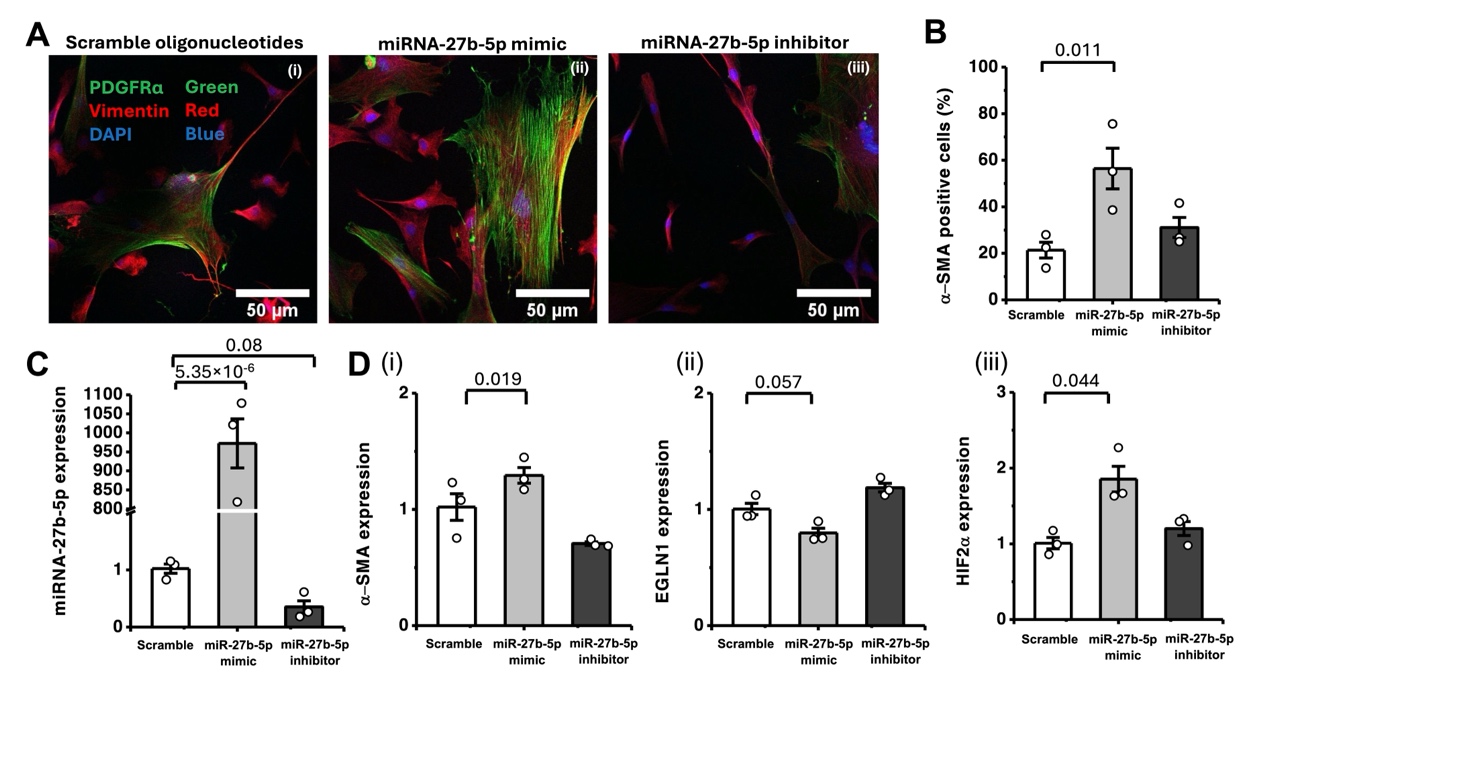
**

**Figure S7. miRNA-27b-5p overexpression and inhibition in human CFs.**

**
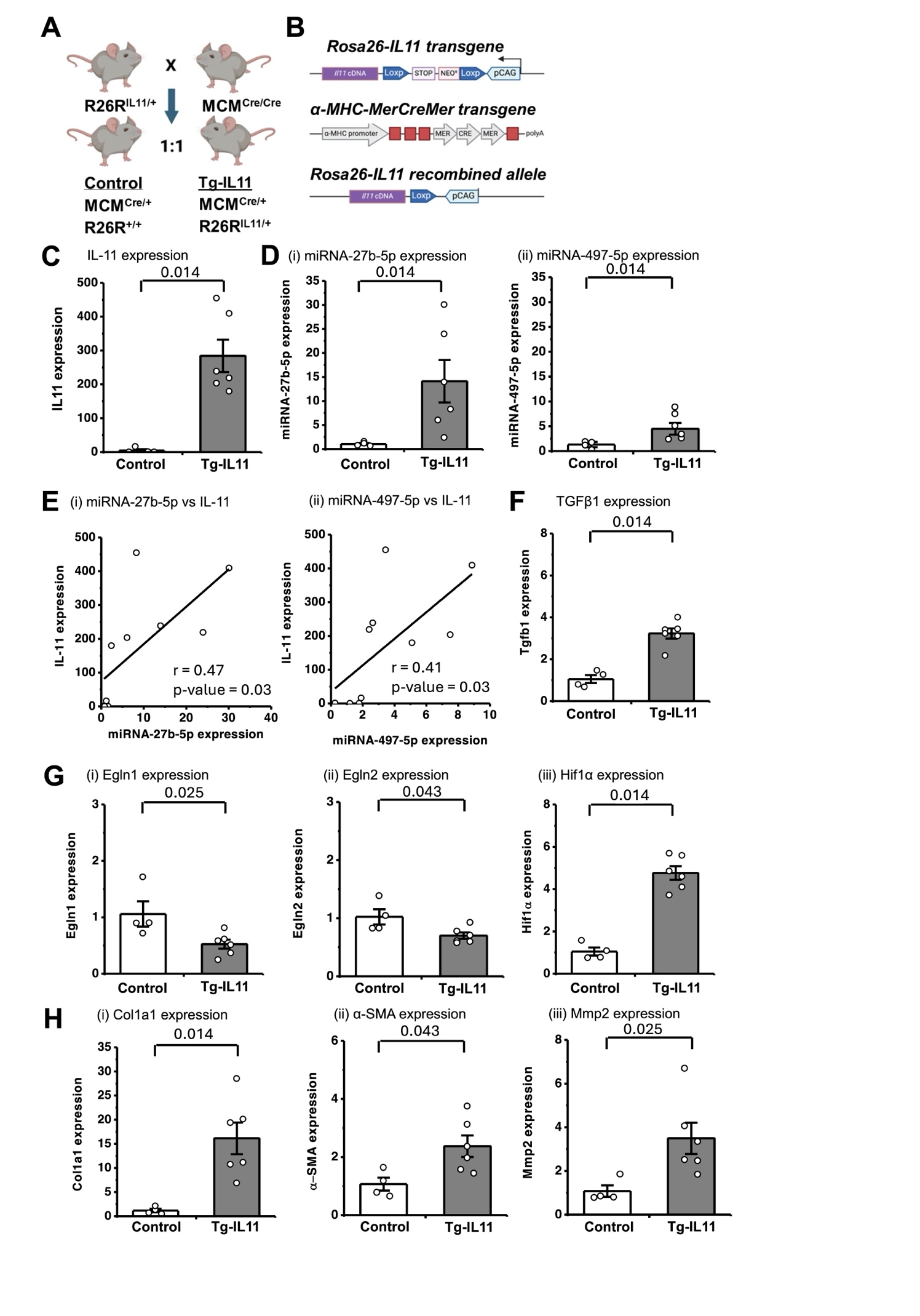
**

**Figure S8. miRNA-27b-5p and miRNA-497-5p are upregulated *in vivo* in** **the heart of Tg-Il11 mice.**


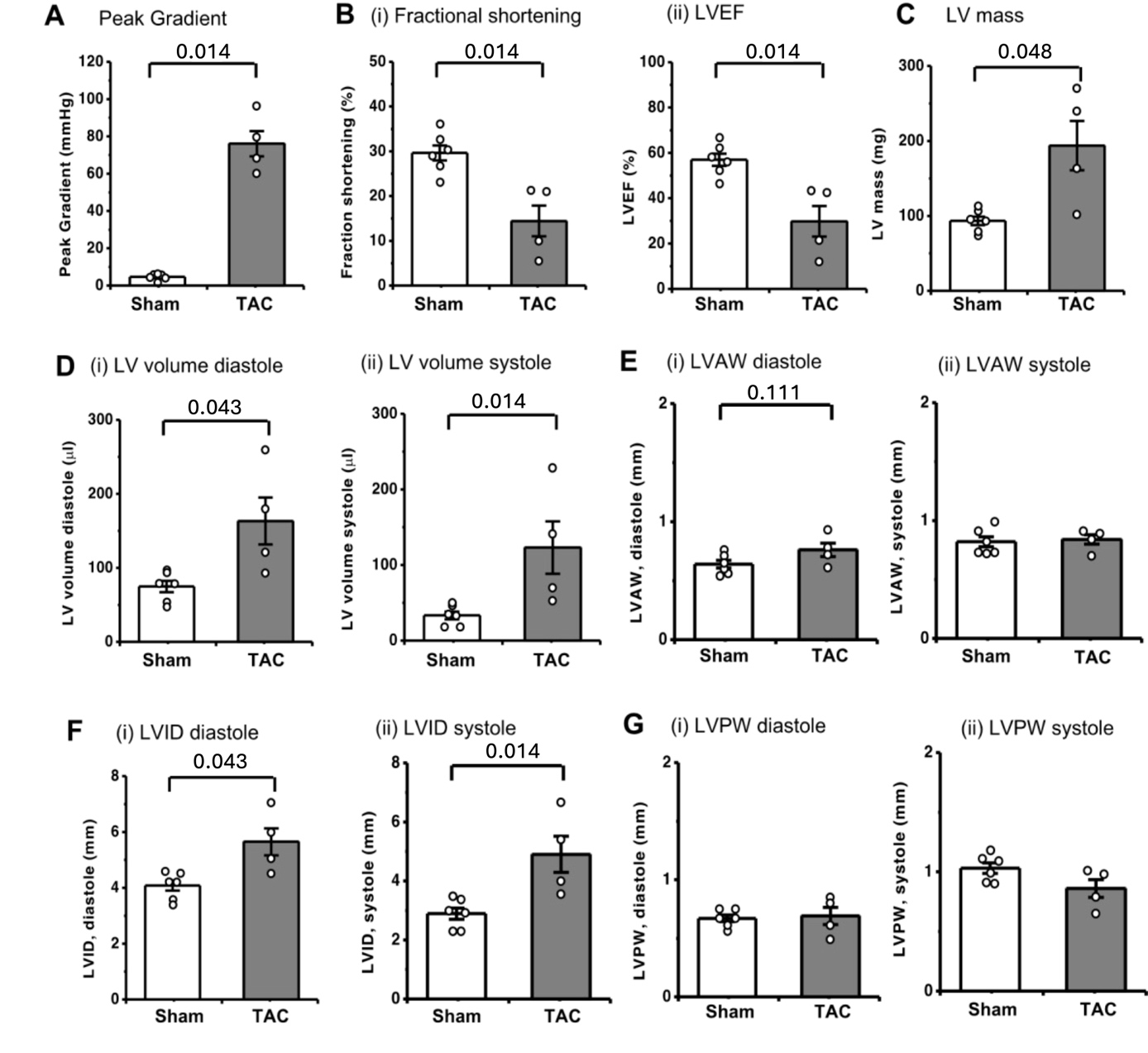


**Figure S9. Cardiac parameters of TAC and sham mice post-surgery.**

**
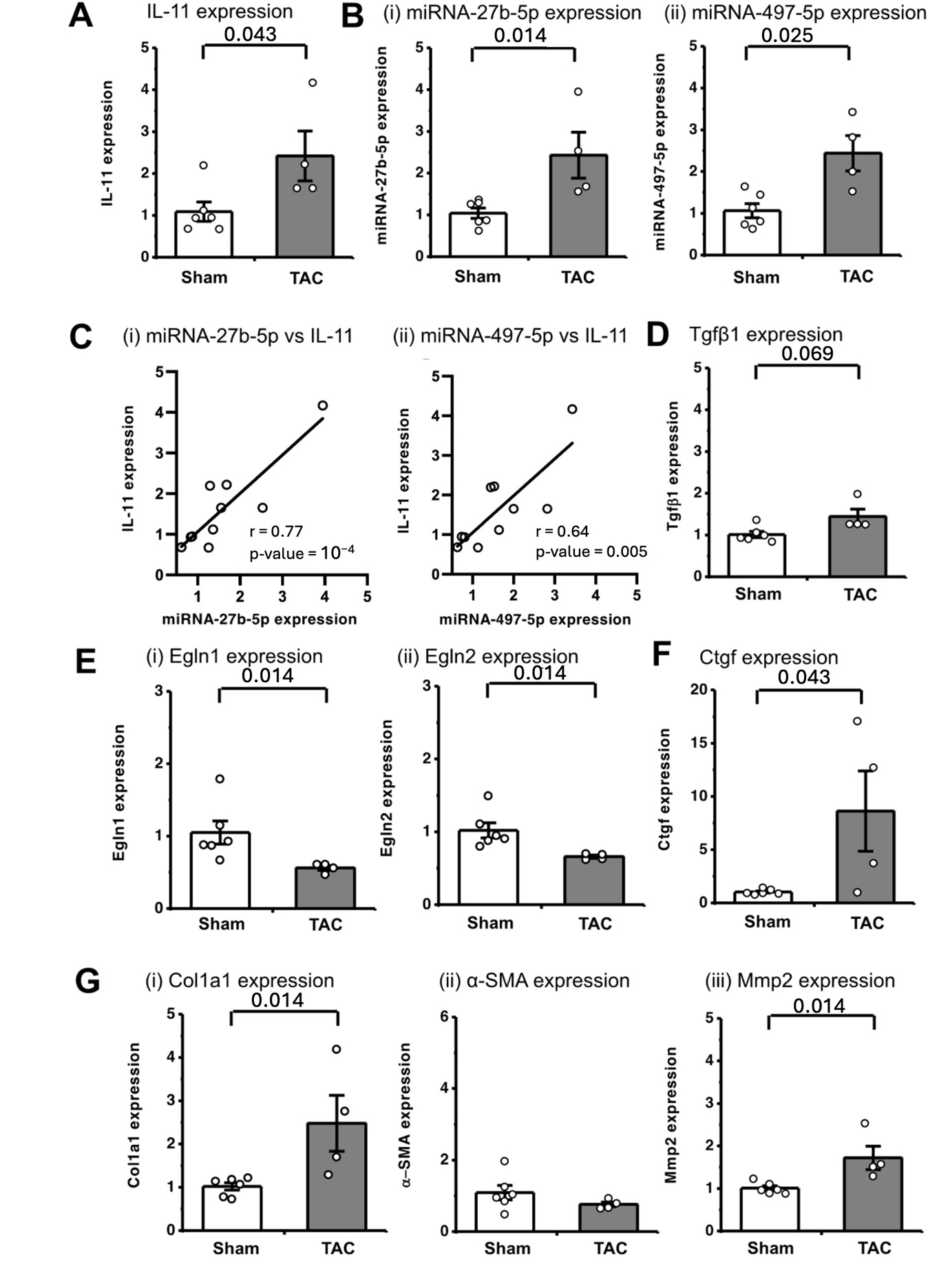
**

**Figure S10. miRNA-27b-5p and miRNA-497-5p are upregulated in the LV of TAC mice.**


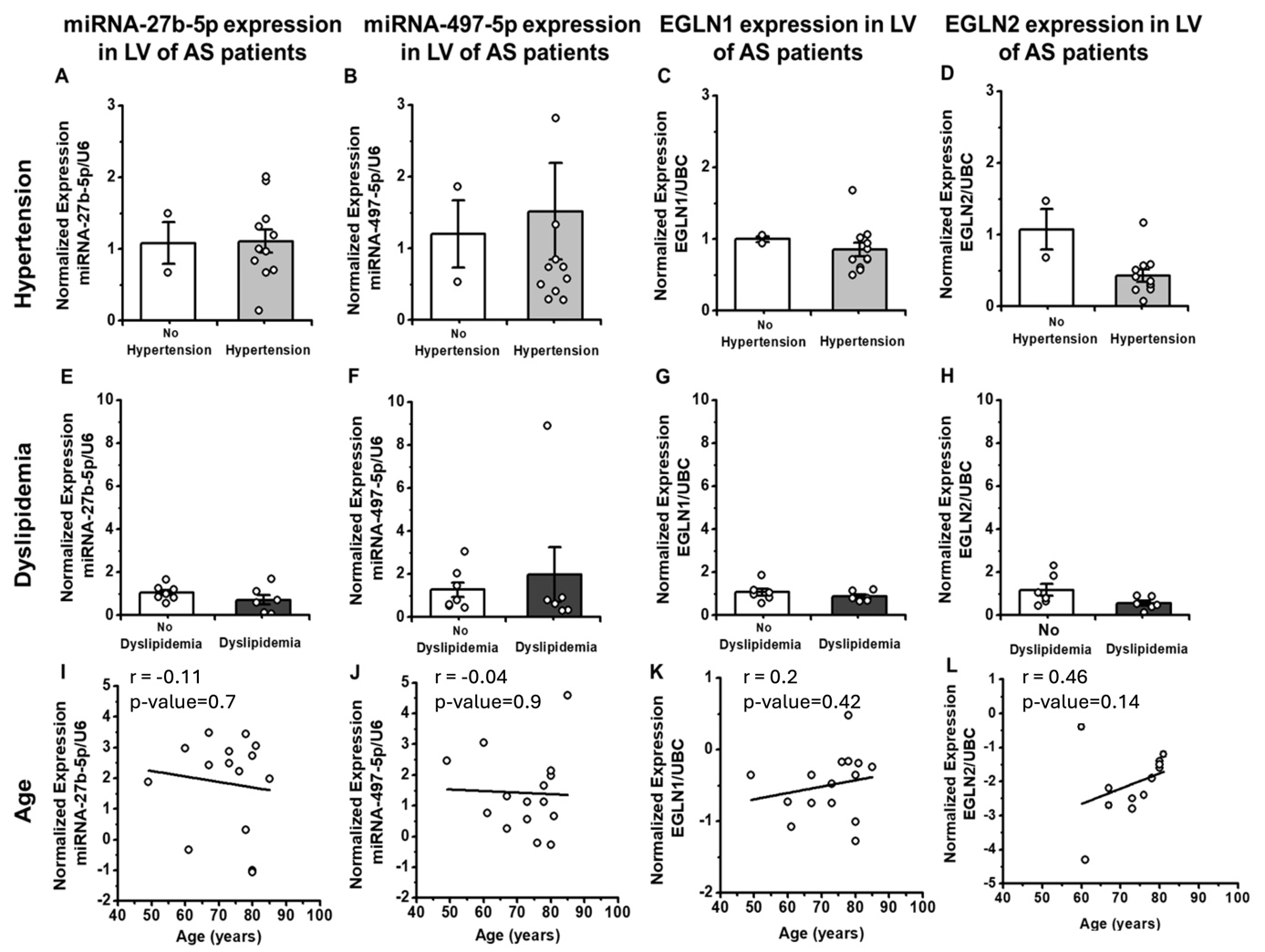


**Figure S11. miRNA-27b-5p, miRNA-497-5p, EGLN1 and EGLN2 expression in LV of AS patients with Hypertension, Dyslipidemia and in old patients.**


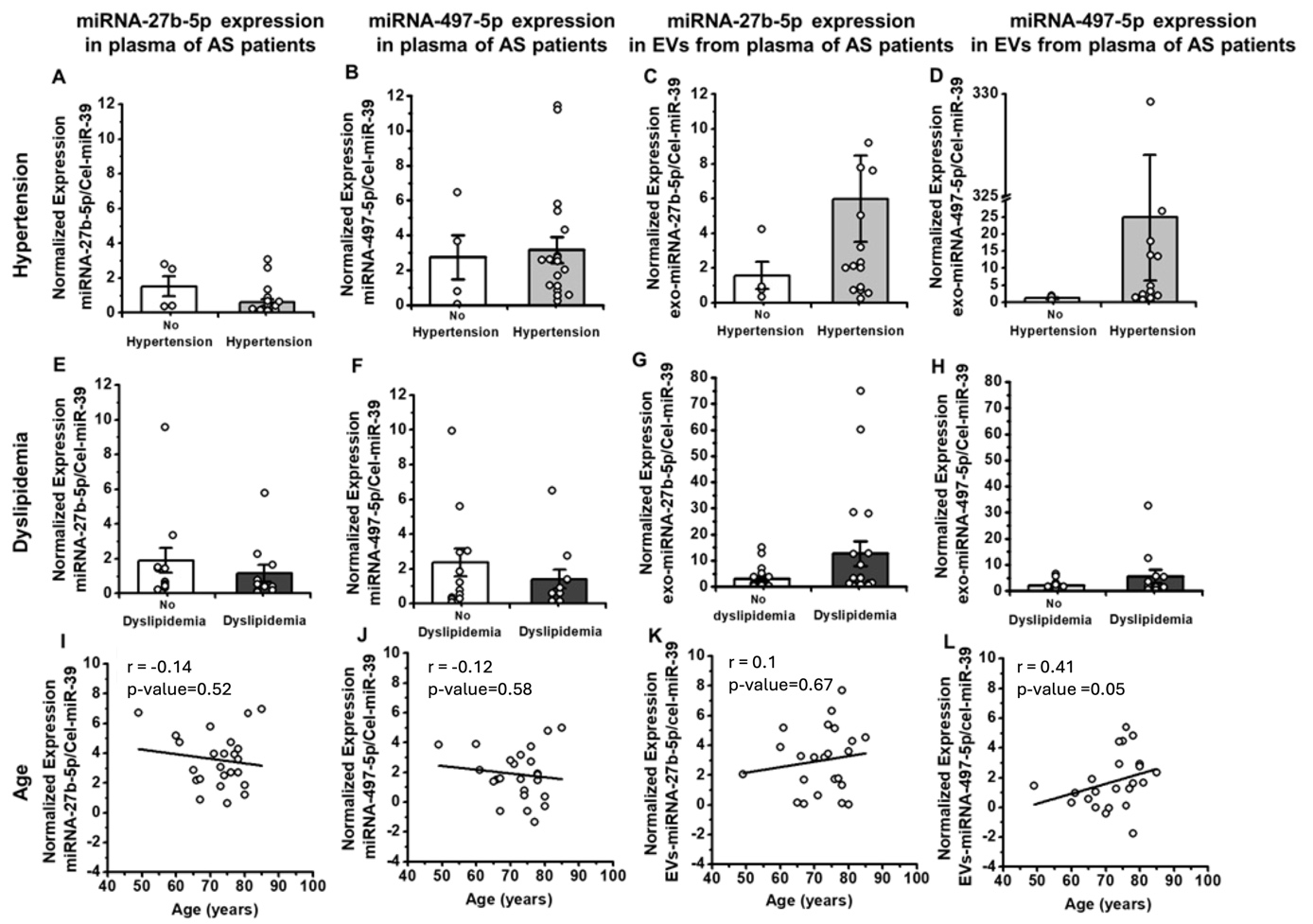


**Figure S12. miRNA-27b-5p and miRNA-497-5p expression in plasma and EVs of AS discovery cohort patients with Hypertension, Dyslipidemia and in old patients.**

**
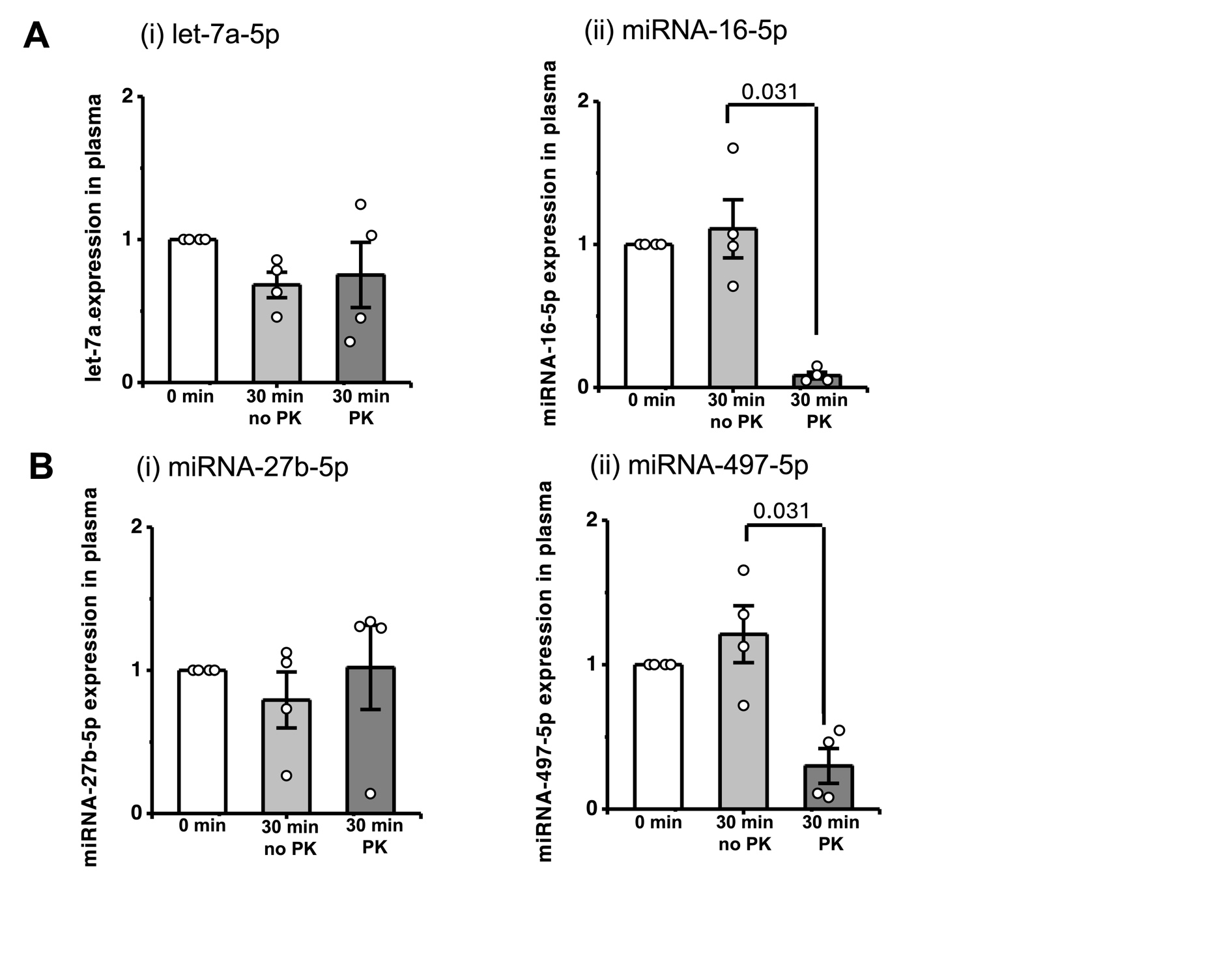
**

**Figure S13. Proteinase K digestion at 55°C selectively sensitizes the plasma non-EV miRNAs to degradation by endogenous RNAses.**

**Supplemental tables**

**Table S1.** Baseline Patient Characteristics: aortic stenosis discovery cohort and healthy donors.

| **Patient Characteristic** | **Aortic stenosis (AS) – discovery cohort** | **Healthy donors** | **p-value** |
| --- | --- | --- | --- |
| **n** | 29 | 35 |  |
| **Age (y)** | 72.2 ± 1.5 | 48.1 ± 3.2 | <0.0001 |
| **Sex (M/F)** | 14/15 | 23/12 | 0.22 |
| **BMI** | 28.4 ± 0.9 |  |  |
| **Hypertension n, (%)** | 23, (79.3%) | 6, (17.1%) | <0.0001 |
| **Dyslipidemia n, (%)** | 14, (48.3%) | 3, (8.6%) | 0.0006 |
| **Smoking history n, (%)** | 4, (13.8%) | 2, (5.7%) | 0.22 |
| **Diabetes n, (%)** | 10, (34.5%) | 3, (8.6%) | 0.05 |
| **Creatinine levels (mmol/L)** | 81.9 ± 4.2 | 105.7 ± 19.6 | 0.82 |
| **Coronary artery disease n, (%)** | 0, (0%) | 0, (0%) | >0.99 |
| **History of arrhythmia n, (%)** | 0, (0%) | 0, (0%) | >0.99 |
| **LVEF (%)** | 59.6 ± 1.4 | 57.7 ± 0.9 | 0.17 |
| **Table 2.** Baseline Patient Characteristics: aortic stenosis discovery and validation patient cohorts. | | | |
| **Patient Characteristic** | **Aortic stenosis (AS) – discovery cohort** | **Aortic stenosis (AS) – validation cohort** | **p-value** |
| **n** | 29 | 14 |  |
| **Age (y)** | 72.2 ± 1.5 | 80.4 ± 2.8 | 0.0017 |
| **Sex (M/F)** | 14/15 | 4/10 | 0.21 |
| **BMI** | 28.4 ± 0.9 | 27.8 ± 1.2 | 0.7 |
| **Hypertension n, (%)** | 23, (79.3%) | 10, (71.4 %) | 0.72 |
| **Dyslipidemia n, (%)** | 14, (48.3%) | 5, (35.7 %) | 0.53 |
| **Smoking history n, (%)** | 4, (13.8%) | 0, (0 %) | 0.28 |
| **Diabetes n, (%)** | 10, (34.5%) | 4, (28.6%) | 0.9 |
| **Creatinine levels (mmol/L)** | 81.9 ± 4.2 | 78 ± 7.1 | 0.45 |
| **Coronary artery disease n, (%)** | 0, (0%) | 0, (0%) | >0.99 |
| **History of arrhythmia n, (%)** | 0, (0%) | 0, (0%) | >0.99 |
| **LVEF (%)** | 59.6 ± 1.4 | 62.4 ± 0.9 | 0.25 |

**Table S3.** AS patient echocardiogram parameters.

| **Echocardiographic parameter** | **Aortic stenosis (AS) discovery cohort** | **Aortic stenosis (AS) validation cohort** |
| --- | --- | --- |
| **Vmax (m/s)** | 4.49 ± 0.3 | 4.72 ± 0.2 |
| **Aortic Valve MG (mmHg)** | 52.9 ± 3.2 | 58.7 ± 7.4 |
| **Aortic Valve PG (mmHg)** | 87.7 ± 6.5 | 90.7 ± 10.3 |
| **Aortic Area (cm2)** | 0.73 ± 0.04 | 0.69 ± 0.06 |
| **LVEF** | 59.59 ± 1.4 | 62.4 ± 0.8 |
| **IVS (mm)** | 14.3 ± 0.5 | 12.8 ± 0.8 |
| **EDVi (ml/m2)** | 54.5 ± 4.9 | 49.1 ± 3.7 |

**Table S4.** Sequences of PCR primers used for mRNA analyses.

| Gene name | Species | amplicon size in base pairs (bp) | Forward primer sequence 5'->3' | Reverse primer sequence 5'->3' |
| --- | --- | --- | --- | --- |
| α-SMA | human | 191 | ATGCTCCCAGGGCTGTTTTCCCAT | GTGGTGCCAGATCTTTTCCATGTCG |
| CTGF | human, mouse | 143 | GTTTGGCCCAGACCCAACTA | GGCTCTGCTTCTCTAGCCTG |
| EGLN2 | human | 79 | AGGGCGGCTGGCACAAA | TAAGGGCTTGGGAAGGGACC |
| EGLN1 | human | 155 | GGCAAAGCCCAGTTTGCTGAC | TTAGCTCGTGCTCTCTCATCTGC |
| GAPDH | human | 131 | GTCTCCTCTGACTTCAACAGCG | ACCACCCTGTTGCTGTAGCCAA |
| HIF1α | human | 170 | GTGAAGACATCGCGGGGACC | GTGGAAGTGGCAACTGATGAGC |
| HIF2α | human | 177 | CACCTCGGACCTTCACCAC | CCGGGACTTCTCCTTCCTCC |
| UBC | human | 199 | GATCGCTGTGATCGTCACTTGACAA | AGTCAGACAGGGTGCGCCCA |
| α-SMA | mouse | 138 | TGCTGACAGAGGCACCACTGAA | CAGTTGTACGTCCAGAGGCATAG |
| Col1a1 | mouse, rat | 176 | CCCAGCCGCAAAGAGTCTAC | CAGGTTTCCACGTCTCACCA |
| Egln1 | mouse, rat | 103 | GAGACCATCGGCCTGCTCAT | CGACCATGGCTTTCGTTCGG |
| Egln2 | mouse | 70 | CTGCTTCTGGCTTGCCTCT | TCTCCTTGTTGCTCCTCAGTG |
| Hif1a | mouse | 110 | CCTGCACTGAATCAAGAGGTTGC | CCATCAGAAGGACTTGCTGGCT |
| Il11 | mouse, rat | 72 | TGGGGACATGAACTGTGTTTGT | GGGGCAACGACTCTATCTGG |
| Mmp2 | mouse, rat | 152 | CCCCGATGCTGATACTGA | CTGTCCGCCAAATAAACC |
| Tgfβ1 | mouse | 107 | TGATACGCCTGAGTGGCTGTCT | CACAAGAGCAGTGAGCGCTGAA |
| Ubc | mouse, rat | 100 | GCCCAGTGTTACCACCAAGAAG | TCACACCCAAGAACAAGCACA |
| α-SMA | rat | 191 | ACCATCGGGAATGAACGCTT | CTGTCAGCAATGCCTGGGTA |
| Adamts5 | rat | 116 | AGTACAGTTTGCCTACCGCC | CGTTAGGTGGGCAGGGTATG |
| Col3a1 | rat | 126 | CCACCCTGAACTCAAGAGCG | ACAGTCATGGGACTGGCATT |
| Gapdh | rat | 77 | TGATTCTACCCACGGCAAGTT | TGATGGGTTTCCCATTGATGA |
| Postn | rat | 178 | TGCAAAAAGACACACCTGCAA | CCGAAGTCAATGGGGCTCTT |

**Table S5.** List of small non-coding RNAs measured with RT-qPCR.

| **miRNA** | **Species** | **Assay ID** |
| --- | --- | --- |
| **rno-miR-27b-5p** | rat | 4440886 |
| **rno-miR-497-5p** | mouse, rat | 001346 |
| **rno-miR-214-3p** | rat | 000517 |
| **rno-miR-16-5p** | mouse, rat, human | 000391 |
| **rno-let-7e-5p** | mouse, rat, human | 002406 |
| **rno-miR-21-5p** | mouse, rat, human | 000397 |
| **rno-miR-351-5p** | rat | 002063 |
| **hsa-miRNA-27b-5p** | mouse, human | 002174 |
| **hsa-miRNA-497-5p** | human | 001043 |
| **U6** | mouse, rat, human | 001973 |
| **cel-miR-39** |  | 000200 |

**Major Resources Table**

In order to allow validation and replication of experiments, all essential research materials listed in the Methods should be included in the Major Resources Table below. Authors are encouraged to use public repositories for protocols, data, code, and other materials and provide persistent identifiers and/or links to repositories when available. Authors may add or delete rows as needed.

**Animals (in vivo studies)**

| **Species** | **Vendor or Source** | **Background Strain** | **Sex** | **Persistent ID / URL** |
| --- | --- | --- | --- | --- |
| Rat | Inotiv | Sprague-Dawley | male | https://www.inotiv.com/researchmodel/hsd-sprague-dawley-sd |
| Mouse | Charles River Laboratories | C57Bl/6N | male | https://www.criver.com/productsservices/find-model/c57bl6mouse?region=3671 |

**Genetically Modified Animals**

|  | **Species** | **Vendor or Source** | **Background Strain** | **Other Information** | **Persistent ID / URL** |
| --- | --- | --- | --- | --- | --- |
| **Parent -**  **Male** | MCM | The Jackson Laboratory | C57BL/6N | Cat:005657 | https://www.jax.org/strain/005657 |
| **Parent - Female** | Rosa26-  Il11 mice | The Jackson Laboratory | C57BL/6N | Cat: 031928 | https://www.jax.org/strain/031928 |

**Antibodies**

| **Target antigen** | **Vendor or**  **Source** | **Catalog #** | **Working**  **concentration** | **Lot #**  **(preferred but not**  **required** | **Persistent ID / URL** |
| --- | --- | --- | --- | --- | --- |
| Collagen I rabbit polyclonal antibody | Abcam Cambridge,  UK | ab34710 | 1:200 (v/v) |  | https://www.abcam.com/en-us/products/primary-antibodies/collagen-icollagen-iii-antibody-  ab34710?srsltid=AfmBOorSngbAvBKwEbXpvfiZahDTzq3AWgFHMzEvpkB XneuPDkslu9nc |
| Alpha-sarco meric actin (α-SMA) clone 5C5 | Sigma  -  Aldrich,  Merck Darmstadt,  Germany | #2172 | 1:500 (v/v) |  | https://www.sigmaaldrich.com/GB/en/search/alpha-smooth-muscleactin?focus=products&page=1&perpage=30&sort=relevance&term=alph a%20smooth%20muscle%20actin&type=product_name |
| PDGF  Rα rabbit  monoclonal  antibody | Abcam Cambridge,  UK | ab  203491 | 1:1000 (v/v) |  | https://www.abcam.com/en-us/products/primary-antibodies/pdgfralpha-antibody-epr22059-270-  ab203491?srsltid=AfmBOor8ltJqeArDSPEH2gOBHZRFez739tiZSshmAhVK 5bsKxlReZf2X |
| α-SMA mouse monoclonal antibody | DAKO | M0851 | 1:250 (v/v) |  | https://www.bioz.com/result/mouse%20monoclonal%20anti%20a%20s mooth%20muscle%20actin%20a%20sma/product/Abcam |
| Vimentin chicken polyclonal antibody | Invitrogen | PA  116759  9 | 1:500 (v/v) |  | https://www.thermofisher.com/antibody/product/Vimentin-AntibodyPolyclonal/PA1-16759 |
| Goat anti- rabbit AlexaFluor  488 | Invitrogen | A11008  08 | 1:250 (v/v) |  | https://www.thermofisher.com/antibody/product/Goat-anti-Rabbit-IgGH-L-Cross-Adsorbed-Secondary-Antibody-Polyclonal/A-11008 |
| Donkey  anti-  mouse  AlexaFluor  488 | Invitrogen | A21202  02 | 1:250 (v/v) |  | https://www.thermofisher.com/antibody/product/Donkey-anti-Mouse-  IgG-H-L-Highly-Cross-Adsorbed-Secondary-Antibody-Polyclonal/A-21202 |
| Goat anti-chicken  AlexaFluor  546 | Invitrogen | A11040  40 | 1:250 (v/v) |  | https://www.thermofisher.com/antibody/product/Goat-anti-ChickenIgY-H-L-Secondary-Antibody-Polyclonal/A-11040 |
| Donkey anti-rabbit  Alexa  Fluor  555 | Invitrogen | A31572  2 | 1:250 (v/v) |  | https://www.thermofisher.com/antibody/product/Donkey-anti-Rabbit-  IgG-H-L-Highly-Cross-Adsorbed-Secondary-Antibody-Polyclonal/A-31572 |
| Goat anti-  Mouse  Alexa  Fluor  488 | Invitrogen | Ab  150121  1 | 1:250 (v/v) |  | https://www.abcam.com/en-us/products/secondary-antibodies/goatmouse-igm-mu-chain-alexa-fluor-488-  ab150121?srsltid=AfmBOorSAjOu4z5aAmKSVd97gAUa3CUgd6pChtwcM 0cKkgYHVKmh2XzI |

| **DNA/cDNA** |  | | |
| --- | --- | --- | --- |
| **Clone Name** | **Sequence** | **Source /**  **Repository** | **Persistent ID / URL** |
| D-001810-10-20 |  | Horizon Discovery | https://horizondiscovery.com/en/gene-  modulation/knockdown/sirna/products/on-targetplussirna-reagents |
| MC11293 |  | mirVana | https://www.thermofisher.com/order/genomedatabase/details/mirna/MC11293 |
| MH11293 |  | mirVana | https://www.thermofisher.com/order/genomedatabase/details/mirna/MH11293 |
| MC20018 |  | mirVana | https://www.thermofisher.com/order/genomedatabase/details/mirna/MC20018 |
| MH20018 |  | mirVana | https://www.thermofisher.com/order/genomedatabase/details/mirna/MH20018 |
| CmiR0001-MR04 |  | GeneCopoeia | https://www.genecopoeia.com/product/search3/# |
| HmiR0145-MR04 |  | GeneCopoeia | https://www.genecopoeia.com/search/microrna/plasm ids/hmir0145-human-hsa-mir-27b-mi0000440-mirnainhibitor-primer |
| HmiR0271-MR04 |  | GeneCopoeia | https://www.genecopoeia.com/search/microrna/plasm ids/hmir0271-human-hsa-mir-497-mi0003138-mirnainhibitor-primer |
| pLUC, #692 |  | Ambion |  |
| pLV[Exp]-CMV> {rEgln1[NM_178334.4] | Egln1 Coding sequence: ATGGCCTCTGACTCCGGAGGACCAGGTGTTTTGTCCGCTTCTGAGAGGGATAGACAGTACTGTGAGCTATGCGGAAAGATGGAAAACCTGCTGCGGTGCGGCAGATGCAGAAGTAGCTTCTACTGCTGCAAGGAGCATCAGAGACAGGATTGGAAGAAGCACAAGCTGGTGTGCCAGGGCGGCGAGGCCCCCAGAGCCCAGCCTGCCCCTGCCCAGCCCAGGGTGGCTCCACCCCCAGGCGGAGCACCCGGCGCCGCCAGAGCCGGAGGCGCCGCCCGGCGAGGAGACAGCTCCACAGCAGCCTCCAGAGTGCCCGGTCCAGAAGATGCCACCCAGGCTGGGTCTGGCCCTGGCCCTGCAGAGCCATCCTCTGAGGATCCTCCTCCTTCTAGGTCCCCCGGCCCTGAACGGGCTAGCCTCTGCCCCGCTGGCGGGGGGCCAGGTGAAGCCCTGTCTCCCTCTGGAGGCCTGAGACCCAACGGCCAGACAAAGCCTCTGCCTGCCCTGAAGCTGGCACTGGAGTACATTGTGCCGTGTATGAACAAGCATGGAATCTGTGTGGTGGATGATTTCCTGGGCAGAGAAACCGGCCAGCAGATCGGCGACGAGGTGCGGGCCCTGCACGACACAGGTAAGTTCACAGACGGACAGCTGGTGAGCCAGAAGTCAGATTCCTCAAAAGACATCAGGGGCGATAAGATCACTTGGATCGAGGGAAAAGAGCCAGGGTGCGAGACCATCGGACTGCTGATGTCCTCCATGGACGATCTGATCCGTCACTGTAGCGGCAAACTGGGCAACTACCGGATCAATGGGCGCACAAAGGCCATGGTGGCCTGCTACCCCGGAAATGGCACCGGGTACGTGCGGCATGTGGACAATCCTAACGGAGACGGAAGATGTGTTACCTGTATCTACTACCTGAACAAGGACTGGGATGCCAAAGTGTCCGGCGGCATCCTGAGAATCTTCCCAGAGGGAAAAGCTCAGTTCGCCGACATCGAGCCCAAGTTCGACAGGCTGCTGTTCTTCTGGAGTGATAGAAGAAATCCACACGAGGTGCAGCCCGCCTACGCCACACGCTACGCTATCACAGTGTGGTACTTTGACGCCGATGAAAGGGCCAGAGCTAAGGTCAAGTACCTGACAGGCGAGAAAGGAGTGCGCGTGGAGCTGAAGCCAAACTCTGTGTCCAAAGACGTGTGA | VectorBuilder | https://en.vectorbuilder.com/vector/VB250616-1495egk.html |

## Cultured Cells

| **Name** | **Vendor or Source** | **Sex (F, M, or unknown)** | **Persistent ID / URL** |
| --- | --- | --- | --- |
| HEK293FT | Invitrogen | Female | https://www.thermofisher.com/order/catalog/product/R70007 |

## Data & Code Availability

| **Description** | **Source / Repository** | **Persistent ID / URL** |
| --- | --- | --- |
| RNAseq on human IL-11 treated CFs | Reilly-O'Donnell B, Ferraro E, Tikhomirov R, et al. Protective effect of UDCA against IL-11- induced cardiac fibrosis is mediated by TGR5 signalling.  Front Cardiovasc Med. 2024;11:1430772. Published 2024 Dec 3. doi:10.3389/fcvm.2024.1430772 | https://pmc.ncbi.nlm.nih.gov/articles/PMC11650366/ |

## ARRIVE GUIDELINES

The ARRIVE guidelines ([https://arriveguidelines.org/)](https://arriveguidelines.org/) are a checklist of recommendations to improve the reporting of research involving animals. Key elements of the study design should be included below to better enable readers to scrutinize the research adequately, evaluate its methodological rigor, and reproduce the methods or findings. **Study Design**

| **Groups** | **Sex** | **Age** | **Number (prior to experiment)** | **Number (after termination)** | **Littermates (Yes/No)** | **Other description** |
| --- | --- | --- | --- | --- | --- | --- |
| Sham C57Bl/6N mice | Male | 8-16 weeks | N = 6 | N = 6 | Yes | Supplementary Methods |
| TAC C57Bl/6N mice | Male | 8-16 weeks | N = 4 | N = 4 | Yes | Supplementary Methods |
| MCMCre/+  R26R+/+ C57Bl/6N mice | Male/Female | 8 – 16 weeks | N = 4 | N = 4 | Yes | Supplementary Methods |
| MCMCre/+  R26R Il11/+ C57Bl/6N mice | Male/Female | 8 – 16 weeks | N = 6 | N = 6 | Yes | Supplementary Methods |
| Sham  Sprague Dawley  rats | Male | 8 – 24 weeks | N = 6 | N = 6 | Yes | Supplementary Methods |
| Coronary artery ligation Sprague Dawley  rats | Male | 8 – 24 weeks | N = 6 | N = 6 | Yes | Supplementary Methods |

**Sample Size:** Animal numbers were estimated a priori and determined from power calculations with 80% power and a 95% confidence interval using standard deviation values from our previously published works for TAC mice (Zaccagnini G, Baci D, Tastsoglou S, et al. miR-210 overexpression increases pressure overload-induced cardiac fibrosis. Noncoding RNA Res. 2025;12:20-33. Published 2025 Jan 31. doi:10.1016/j.ncrna.2025.01.009) for MCMCre/+ R26R Il11/+ C57Bl/6N mice (Sweeney M, O'Fee K, Villanueva-Hayes C, et al. Cardiomyocyte-Restricted Expression of IL11 Causes Cardiac Fibrosis, Inflammation, and Dysfunction. Int J Mol Sci. 2023;24(16):12989. Published 2023 Aug 20. doi:10.3390/ijms241612989) and coronary artery ligation rats (Reilly-O'Donnell B, Ferraro E, Brody R, et al. Protective effect of ursodeoxycholic acid upon the post-myocardial infarction heart. Cardiovasc Res. Published online September 12, 2025. doi:10.1093/cvr/cvaf133); for MI, sham and healthy rat (Reilly-O'Donnell B, Ferraro E, Tikhomirov R, et al. Protective effect of UDCA against IL-11- induced cardiac fibrosis is mediated by TGR5 signalling. Front Cardiovasc Med. 2024;11:1430772. Published 2024 Dec 3. doi:10.3389/fcvm.2024.1430772; Lyon, A. R., MacLeod, K. T., Zhang, Y., Garcia, E., Kanda, G. K., Lab, M. J., Korchev, Y. E., Harding, S. E., & Gorelik, J. (2009). Loss of T-tubules and other changes to surface topography in ventricular myocytes from failing human and rat heart. Proceedings of the National Academy of Sciences of the United States of America, 106(16), 6854–6859. doi:10.1073/pnas.0809777106).

## Inclusion Criteria

The inclusion/exclusion criteria set a priori was that any animal demonstrating sustained changes in behavior, surgical complications, or other adverse effects resulting from the surgery or tamoxifen induction. If the animal shows signs of the distress they were humanely culled and excluded from analysis.

## Exclusion Criteria

The inclusion/exclusion criteria set a priori was that any animal demonstrating sustained changes in behavior, surgical complications, or other adverse effects resulting from the surgery or tamoxifen induction. If the animal showed signs of the distress, they were humanely culled and excluded from analysis.

**Randomization**

Animals were housed in a random order on shelves. Animals were randomised into control group or surgery group using a simple randomization ‘numbers-out-of-a-hat’ method.

## Blinding

Investigators performing qPCR analyses were initially blinded to experimental group allocation, with group identities revealed during data analysis. Microscopy-based image analyses were performed on blinded datasets, with unblinding only after completion of image processing and data analysis.
